# Chewing through the Miocene: an examination of the feeding musculature in the ground sloth *Hapalops* from South America (Mammalia: Pilosa)

**DOI:** 10.12688/f1000research.3282.1

**Published:** 2014-04-04

**Authors:** Virginia L. Naples, Robert K. McAfee

**Affiliations:** 1 Department of Biological Sciences, Northern Illinois University, Illinois, 60115, USA; 2 Department of Biological & Allied Health Sciences, Ohio Northern University, Ada, Ohio, 45810, USA

## Abstract

*Hapalops*, a smaller-sized and early sloth of the Megatheroidea, appeared in the middle Miocene Santa Cruz formation of Argentina. This genus is part of the group from which later, larger megatheroids arose, i.e., *Nothrotheriops* and *Megatherium*. Many cranial characters support this idea; however *Hapalops* is not merely a smaller antecedent of the later forms. Specifically, *Hapalops* retains short anterior caniniform teeth, and a temporomandibular joint elevated above the cheek tooth row; a combination distinct among sloths. An elevated temporomandibular joint occurs in *Bradypus*, a tree sloth with anterior chisel-shaped teeth instead of caniniforms, and the tree sloth *Choloepus, *which is aligned with the megalonychids, has anterior caniniforms. *Hapalops* has an elongated zygomatic ascending process that is reminiscent of that in *Bradypus; *however, the *Bradypus* skull is extremely foreshortened while that of *Hapalops* is elongated, as in nothrotheres, but not deepened as in megatheres. Previous work identified many sloth cranial character complexes, and functional limitations on skull feature combinations. The unique *Hapalops* character patterns indicate a selective feeder with a mediolaterally oriented grinding stroke during mastication.

## Introduction

The Xenarthra has two divisions, the Cingulata and the Pilosa. The cingulates have carapaces, i.e., the extinct glyptodonts and giant armadillos, and the living armadillos. Pilosans instead are covered with hair, although some retain bony dermal ossicles. This group contains sloths and anteaters with strikingly different body forms and habits. Four sloth families have extinct representatives, the Mylodontidae, the Megalonychidae, the Megatheriidae and the Nothrotheriidae, and one has only extant members, the Bradypodidae. The anteaters are united in a single family, the Myrmecophagidae ( Scott, 1937; Simpson, 1945; McKenna & Bell, 1997).

Many early sloths were small and appeared in the Oligocene already as obligate herbivores, albeit retaining morphologic influences from insectivorous or myrmecophagous antecedents. Myrmecophagy specializations in sloth progenitors have been suggested as restricting later group members from developing a feeding apparatus typical of other herbivores ( Winge, 1941; Hirschfeld & Webb, 1968; Patterson & Pascual, 1972; Hirschfeld, 1976; Webb, 1985). Some more unusual herbivore characters inherited by sloths are: (1) narrow, greatly elongated skulls, (2) zygomatic arches missing the anteroposterior jugal-squamosal temporal process connection, (3) decreased numbers and types of teeth, (4) simplified shapes for retained teeth, (5) enamel loss, (6) ever-growing teeth, (7) fewer anterior teeth, and (8) a small buccal opening. All of these characters, advantageous to myrmecophages, are disadvantageous to the typical herbivore condition. Another important character inferred in extinct sloths because of their large hyoid apparatuses ( Naples, 1987; Naples, 1989), and by comparison with Recent tree sloths ( Naples, 1986) and xenarthran anteaters, are large, long and highly mobile tongues which are also characteristic of insectivorous forms ( Owen, 1862; MacAlister, 1875; Reeve, 1940; Montgomery, 1985; Naples, 1999).

Xenarthrans also vary widely in cranial specializations, perhaps reflecting bulk versus selective feeding strategies. Characters typifying bulk feeding, as in *Lestodon* and *Glossotherium,* include a wide a muzzle and predental spout. Sloths considered selective feeders have narrow muzzles and predental spouts longer than wide, such as the scelidotheres ( Bargo *et al.*, 2006a; Bargo *et al.*, 2006b). Further specialization for aquatic feeding is noted for *Thalassocnus* (Muizon *et al.*, 2004).

In spite of an odd herbivore morphology, the combined structural and functional features among sloth lineages succeeded sufficiently to permit small sloths to evolve into moderate and large forms. Megatheroids display this tendency most strikingly, with genera ranging from small (sheep-sized) early genera such as *Hapalops*, to moderate (bear-sized) forms such as *Nothrotheriops,* and finally, to the giant (elephant-sized) megatheres such as *Megatherium* and *Eremotherium*. Recent tree sloths are obligate suspensory arboreal folivores, whereas extinct sloths are considered “ground” sloths. The unique inverted tree sloth locomotion pattern has been cited as evidence of a close phylogenetic relationship between these genera ( Webb, 1985). However, uniting the tree sloths in Bradypodidae is now viewed as incorrect, with each genus taxonomically distinct at the family level ( Patterson & Pascual, 1972; Webb, 1985; Gaudin, 2004). As such, *Bradypus* is retained within Bradypodidae while *Choleopus* is in Megalonychidae. Extinct sloths have been divided into four families, Megalonychidae, Megatheriidae, Nothrotheriidae and Mylodontidae, although these associations partially assume that all are terrestrial ( Gaudin & McDonald, 2008). However, some of the small sized extinct species including *Hapalops,* may have been at least partially arboreal ( White, 1993; Pujos *et al.*, 2007). Larger sloths, such as *Nothrotheriops,* might have been semiarboreal, or as capable of climbing as are bears. Perhaps the young of even the largest species could climb. The living South American Andean spectacled bear, *Tremarctos ornatus*, may weigh up to 80 kilograms. It is the most arboreal ursid, living mostly in trees, including building nests and sleeping arboreally ( Nowak, 1999). Other authors also suggest that some extinct sloths might have been arboreal ( Anthony, 1916; Matthew & Paula Couto, 1959; Romer, 1966; Hirschfeld & Webb, 1968; Paula Couto, 1971; Mayo, 1980; Scillato-Yane, 1980; Coombs, 1983; Sherman, 1984; Hirschfeld, 1985; Webb, 1985; White, 1993; Pujos *et al.*, 2007).

Many mammalian insectivorous or omnivorous basal stocks evolved specialized lineages; therefore, it is unsurprising that at least one xenarthran group gave rise to herbivorous forms ( Winge, 1941; Janis *et al.*, 1998). However, fossil record gaps prevent direct observation of such changes, and the four fossil sloth families are distinct from their earliest appearance. Lacking direct evidence, reconstruction of morphologies that allowed sloth divergence may be modeled by study of evolutionary trends in known xenarthran lineages. Goals of this study include identification of related structural characters, their association into functional complexes, and evaluation of the potential feeding habits of *Hapalops*.

## Materials and methods

Studies of *Hapalops* are often hampered by taxonomic issues as over twenty species were historically assigned to the genus (De Iuliis and Pujos, 2006). Many such species were created with little diagnostic description or character explanation (Ameghino 1889; Scott, 1903–1904; Hirschfeld, 1985). Therefore, a contemporary taxonomic revision will likely result in a reduced number of *Hapalops* species. As review and revision of the systematics of *Hapalops* and closely related taxa is beyond the scope of this paper, only currently identified generic characters are recognized here. Typical *Hapalops* species features were grouped to establish an overall generic level character suite. Anatomical reconstructions were also created as a means of studying other extinct sloths ( Naples, 1987; Naples, 1989; Naples, 1990; Muizon *et al.*, 2004; Bargo *et al.*, 2006b), especially when complete specimens were unavailable. Additionally, the variability among *Hapalops* specimens studied here is less than what is considered intraspecific variation among anatomical characters in a single living species.

Specimens examined were skulls and jaws of *Hapalops* from the Field Museum of Natural History, Department of Geology, the Museum of Natural History at the University of Kansas, and the American Museum of Natural History. All specimens ( Table 1) were considered “adult”, based on partial sutural fusion, cortical bone density, and being in the largest size range ( Naples, 1982; Anderson & Handley, 2001). Cranial scars indicating origin and insertion of facial, masticatory, lingual, pharyngeal, cervical, intrinsic and extrinsic back muscles were examined.

**Table 1.** *Hapalops* specimens examined for this study. The names of museums are represented by the following acronyms: American Museum of Natural History (AMNH), Field Museum of Natural History (FMNH) and University of Kansas Natural History Museum (KU).

Museum	Genus and species name	Specimen number	Elements examined
AMNH	*Hapalops brachycephalis*	9176	Skull
AMNH	*Hapalops rectangularis*	9222	Mandible
FMNH	*Hapalops reutmeyeri*	P13141	Skull
FMNH	*Hapalops elongatus*	P13138	Skull
FMNH	*Hapalops elongatus*	P13145	Skull
FMNH	*Hapalops elongatus*	P13133	Skull and mandible
FMNH	*Hapalops elongates*	P13121	Mandible
FMNH	*Hapalops sp.*	P13278	Skull
FMNH	*Hapalops sp*.	P13122	Skull and mandible
FMNH	*Hapalops sp.*	P13136	Mandible
FMNH	*Hapalops reutmeyeri*	P13130	Mandible
FMNH	*Hapalops longiplatus*	P13143	Mandible
KU	*Hapalops reutmeyeri*	KU 145120	Skull and mandible
YPM	*Hapalops longiceps*	15523	Skull and mandible

Study of soft tissues of living species allows modeling of patterns in related extinct species, permitting a hypothetical soft tissue reconstruction, and therefore, an estimate of the appearance and function of tissues of fossils. Tree sloth muscles, which show convergent patterns, were identified by tracing innervations during dissection of preserved specimens of *Bradypus* and *Choloepus* ( Naples, 1985a; Naples, 1985b). As the closest living relatives to extinct sloths, we use them to infer similar muscle arrangement patterns despite their disparate phylogenetic associations ( Gaudin, 2004). Terminology agrees with prior studies of tree sloths and fossil sloths ( Naples, 1982; Naples, 1986; Naples, 1987; Naples, 1989), as well as the Nomina Anatomica Veterinaria (2005). Muscle and muscle segment names have been homologized with those in the older literature, and references to muscle heads or slips indicate origins and insertions, respectively, for muscles composed of more than one part or belly.

Additionally, dental occlusion, wear and mandibular movement patterns were determined and compared to those of *Bradypus*, *Choloepus*, *Eucholaeops*, *Nothrotheriops* and *Thalassocnus* ( Naples, 1982; Naples, 1985a; Naples, 1987; Naples, 1995; Muizon *et al.*, 2004; Bargo *et al.*, 2009). No *Hapalops* crania or mandibles were sufficiently complete or uncrushed to allow articulation, and few cranial and mandibular specimens were unequivocally associated. Therefore, the illustrations have been drawn as composites, approximating cranial feature relationships as they would have been in the intact sloth. All illustrations were drawn by one of the authors (VLN).

## Results

### Cranial osteology

Sloth crania differ from those of other xenarthrans in having: (1) reduced edentulous premaxillae that may retain unfused sutures even in aged adults, (2) elongated maxillae, (3) zygomatic arches with elaborated ascending and descending jugal processes, (4) pterygoid bones elongated into flanges or inflated sinuses, (5) fused mandibular symphyses lacking sutural traces, and (6) an elongate predental spout. These characters show different forms, and not all occur in all sloths. Nevertheless, previous sloth cranial studies ( Naples, 1982; Naples, 1987; Naples, 1990) showed characters associated into complexes or suites. Although *Hapalops* shows many such sloth synapomorphies, this genus also displays unique character complexes indicative of the transitions necessary to revert to an herbivorous lifestyle.

### Dorsal and lateral view characters

**Figure 1.**
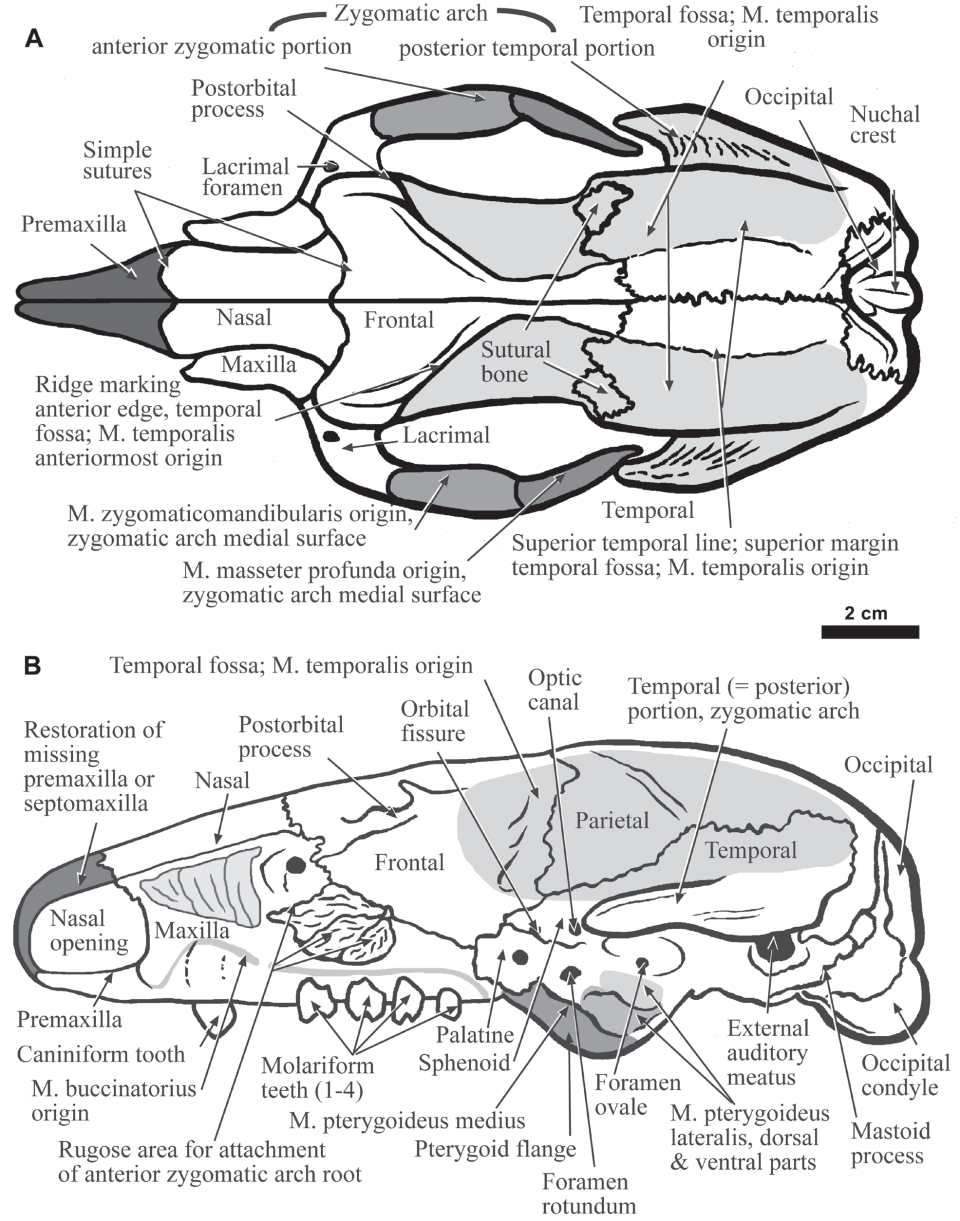
The skull of *Hapalops*, dorsal view ( **A**) and lateral view ( **B**), with skeletal and dental features discussed in the text labeled. The scars of muscle origin are shown by shaded areas. The anterior portion of the zygomatic arch has been removed from **B** to show the rugosity of the articular surface, and to reveal structures on the side of the skull that would otherwise be hidden.

**Figure 2.**
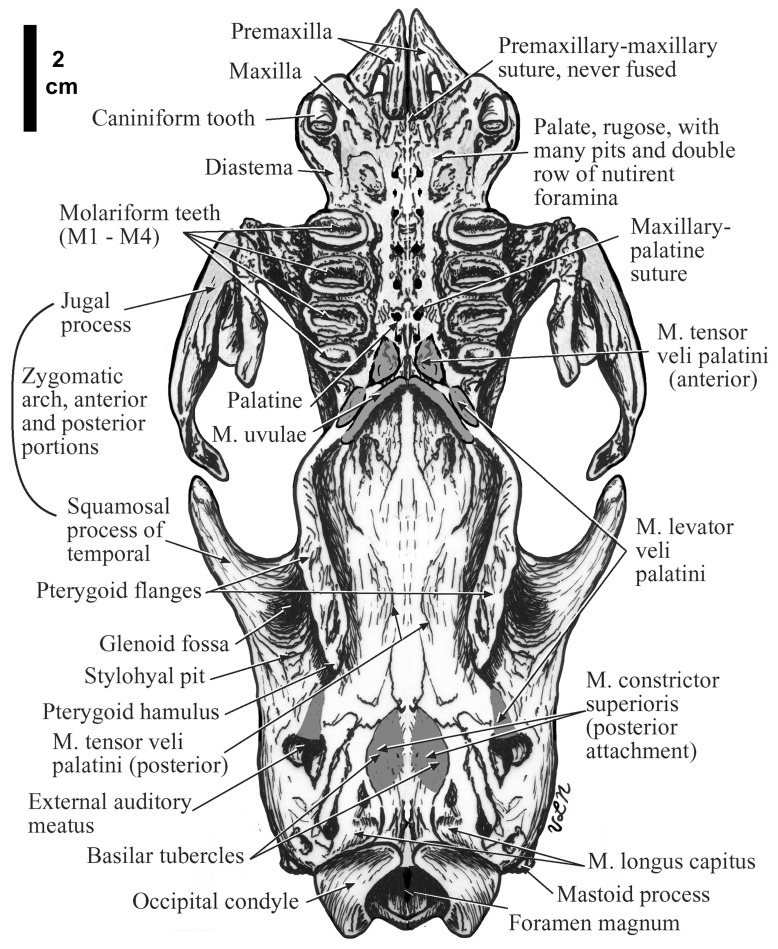
The ventral view of the skull of *Hapalops* is shown with osteolgical and dental features discussed in the text labeled and the scars of origin and insertion of muscles shaded.

**Premaxilla—**Sloth skulls show great shape diversity, but in the earliest genera, as well as some later forms, they tend to be elongate, as is the case for *Hapalops* ( Figure 1, Figure 2). Premaxillae are rarely preserved and, even if present, generally not *in situ*, although one *Hapalops reutmeyeri* specimen (KU 145120) shows intact premaxillae loosely articulated to the maxillae. Dorsally, these bones are narrow, elongate, pointed anteriorly, and perforated by a premaxillary foramen. They widen posteriorly to equal the nasals at the dorsal premaxillary-nasal suture, characterized by a simple, straight abutting joint. Sloths have sometimes been restored with a bony extension forming a nasal ring from the premaxillae dorsally to the nasals ( Wible *et al.*, 1993; Bargo *et al.*, 2006b); confirmed in *Mylodon* [= *Grypotherium*] *darwini*, a sloth with very large nasal openings ( Reinhardt, 1879). No specimen we examined has this area preserved. In *Hapalops* the anterior ends of the ventral, horizontal portion of the premaxillae are shallow, and curve upward ( Figure 1B). They are slightly rugose, indicating the presence of an anterovertical nasal extension and large nasal openings, typical of all sloths and anteaters ( Naples, 1982; Naples, 1985a; Naples, 1999). The interpremaxillary sutures remain unfused, even in adults where most other sutures are fused, and in some cases, at least partially obliterated. Extreme premaxillar slenderness contributed to distortion of available specimens; therefore, these bones have been idealized in illustrations of the reconstructed skull ( Figure 1, Figure 2).

The premaxilla is attached only to the palatal and slightly to the lateral portions of the maxilla, although the missing vertical portion, sometimes also termed a septomaxilla, is represented by a shaded area in Figure 1B ( Wible *et al.*, 1993). The skull slopes gently posterodorsally from the nasals, unaltered by the bulges for the anterior zygomatic arch root and the postorbital process. A slight dorsal parietal swelling in some specimens allowed for a larger braincase. The occiput is gently rounded, the occipital condyle profile round, and the condyles almost entirely ventral to the skull.

**Nasal—**Dorsally ( Figure 1A), the nasal is an elongate rectangle, flared slightly posteriorly with the widening of the anterior zygomatic arch root and slightly pointed anteriorly. The anterior edges are roughened, as for attachment of a bony premaxilla and/or septomaxilla. Posteriorly, the nasofrontal suture is curved, simple and convex, and lack the interdigitating complexity that increases surface area in most sutures to allow stronger bone-bone junctions ( Herring, 1972; Herring & Teng, 1995). Anteriorly each shows a convex mediolateral curve and slight depression of the internasal suture.

**Frontal—**This bone approximates a rectangle dorsally ( Figure 1A), and the simple, straight interfrontal suture remains unfused and visible in specimens of all ages. Anteriorly, the frontals flare broadly to form the anterior origin for the zygomatic arch. Further posteriorly, a second frontal bulge forms the short, rounded overhanging postorbital process bearing an anteroposteriorly elongated depression housing the large and obvious cranial foramina, including the foramen rotundum, sphenoidal fissure and the optic canal ( Figure 1B). In some specimens, a prominent ridge posterior to the postorbital process makes a broadly curved “V” with the apex pointing posteriorly. This shape forms the anterior margin of the extensively variable M. temporalis origin ( Figure 1). In some specimens, the “V” points posteriorly along the parietals, producing an elevated sagittal table. In other specimens, a narrow sagittal crest occurs even though the “V”-shaped ridge does not. Alternately, there is no sagittal crest. These differences reflect the relative degree of development of the anterior limit of the temporal fossa, and therefore, the anterior extent of the M. temporalis. Such differences may result from age or sex differences, or possibly species level differences.

**Parietal—**This bone curves in a gentle convexity for the laterally bulging braincase, the widest skull region in dorsal view ( Figure 1A). The parietal-temporal suture shows a raised ridge, and the typical convoluted mammalian pattern ( Figure 1B). Laterally, the bone bulges posterodorsally. Most of the temporal fossa, *i.e.* the M. temporalis origin, is from the outer temporal surface, marking the dorsal boundary of muscle’s origin. Animals with a farther lateral superior temporal line are probably younger, even though they may be adult in size and overall skull shape ( Naples, 1982; Anderson & Handley, 2001).

**Occipital—**This bone is visible dorsally, and the parieto-occipital suture is complex and “C”-shaped, with a posterior facing open end. Dorsally, the occipital condyles are invisible ( Figure 1A). This bone bears markings for muscles originating on the prominent nuchal crest, *i.e.* those that control the position of the head on the neck.

**Maxilla—**The bone forms much of the skull anterolaterally, and bears the upper dentition ( Figure 1, Figure 2). It has a rounded, rugose lateral projection with many pits and projections that attach to the jugal bone. The maxillary-frontal suture is complex.

**Lacrimal—**This bone is a prominent and rounded, rectangular shape on the lateral skull surface, ventral to the nasal and frontal ( Figure 1). It has a prominent lateral-facing lacrimal foramen, a feature shared by sloths and anteaters, but not obvious in other xenathrans or other mammalian orders.

**Figure 3.**
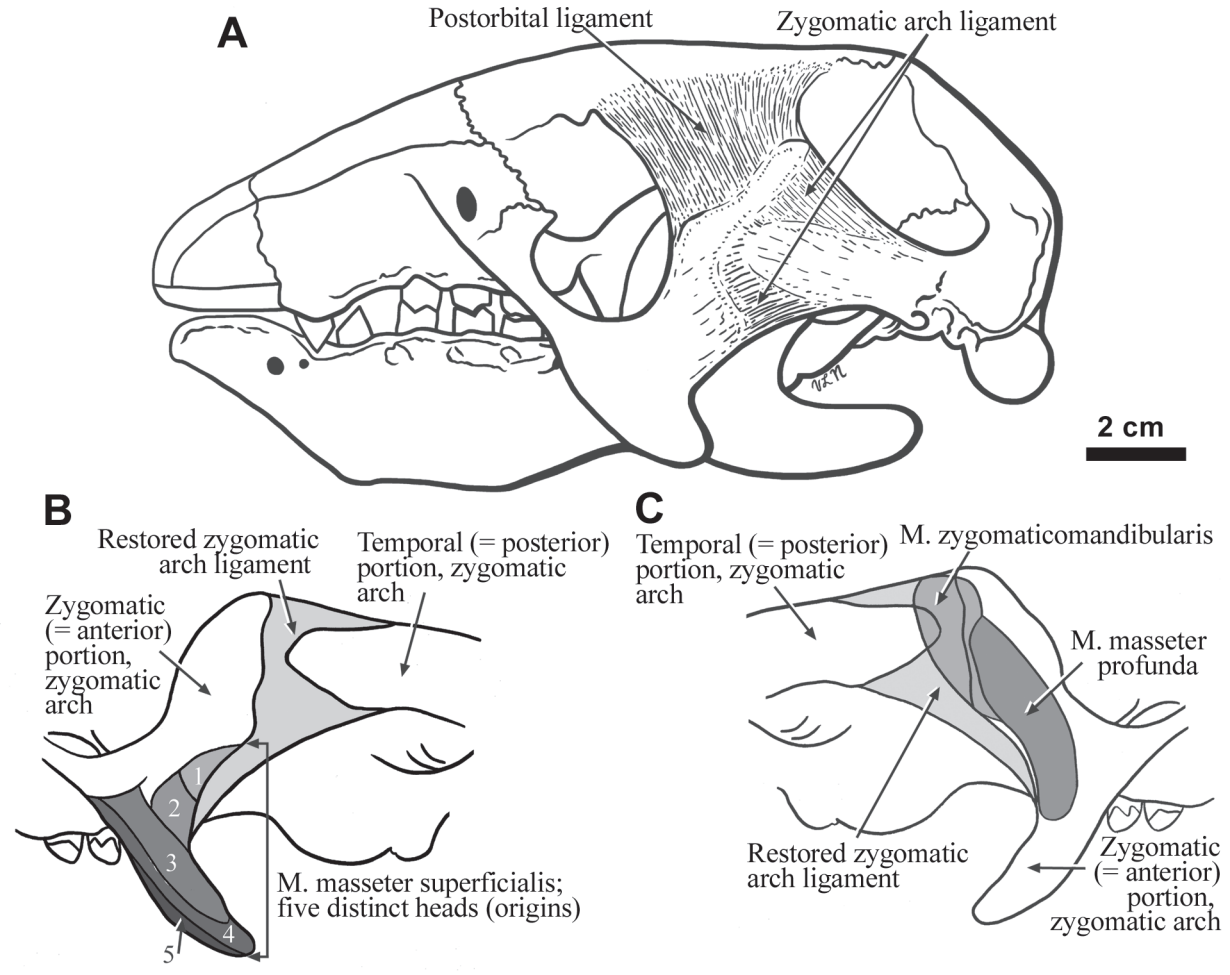
The cranium of *Hapalops* in lateral view showing the restored cranial ligaments ( **A**). Enlargements of the lateral ( **B**) and medial ( **C**) surfaces of both the anterior and posterior portions of the zygomatic arch in *Hapalops* are also shown, along with the zygomatic arch ligament, and the locations of the scars of muscle and muscle head origins that are indicated by differentially shaded areas.

**Jugal (= Malar, Zygomatic)—**This bone forms the anterior part of the zygomatic arch ( Figure 1, Figure 3). Many specimens examined here, as is common in fossil sloths, lack the anterior zygomatic arch root. This large and slender projection is vulnerable to breakage during fossilization, and the maxillolacrimal-jugal suture never fuses, even in the oldest and largest adults. Because this connection is weak, the entire jugal is typically lost. In contrast, in most mammals, the maxillolacrimal-jugal suture fuses, and therefore a portion of the anterior root of the zygomatic arch usually remains attached to the skull, even if the middle region is broken, crushed or missing.

The zygomatic arch lacks an anteroposterior bony connection in *Hapalops*, as in the living tree sloths *Bradypus* and *Choloepus*, and some of the smaller extinct genera [( Matthew & Paula Couto, 1959; Paula Couto, 1971; Naples, 1982) Figures 1A; 3]. In living forms, the anterior portion is bound to the posterior ( *i.e.,* the anteriorly projecting process of the temporal bone) by a ligamentous sheet; therefore a similar connecting ligament has been restored in *Hapalops*. The jugal in this sloth shows both dorsal and ventral processes. The dorsal process is elongate and posterodorsally oriented, as in *Bradypus*. The ventral process has lateral rugosities and depressions for origin of the M. masseter superficialis heads ( Figure 3B). The medial zygomatic surface is rugose, with several depressions, reflecting the origin of the M. masseter profunda and the M. zygomaticomandibularis ( Figure 3C). The latter likely also arose, in part, from the anteroposterior connecting ligament, as well as from an anteromedial depression of the anteriorly projecting squamosal process of the temporal. This arrangement allows the zygomatic arch to act as a single functional unit, as in other mammals.

**Sphenoid—**This bone is often considered to consist of several elements, including orbitosphenoid, alisphenoid, presphenoid and basisphenoid ( Wible & Gaudin, 2004). However, in *Hapalops*, all of these fuse, or are indistinguishable in available specimens. In juvenile tree sloths these bones are distinct, with the orbito- and alisphenoid visible laterally, and the pre- and basisphenoid visible ventrally. The composite bone occupies the ventral portion of the lateral skull wall, including the orbit posteriorly and the anterior and ventral portion of the temporal fossa. It meets its opposite at the ventral midline posterior to the choanae. The four major foramina of the orbital region pass through this bone ( Figure 1B).

**Palatine—**This bone forms the anterior portion of a thin flange extending ventrally, which is completed posteriorly by the pterygoid and dorsally by the orbito- and alisphenoid components of the composite sphenoid ( Figure 1B, Figure 2). In previously published literature, these projections have been designated as pterygoid flanges, because those bones constitute the greater portion of this feature ( Parker, 1885; Naples, 1982; Gaudin, 2004).

**Pterygoid—**This bone extends ventrally as a thin flange posteriorly from the anterior palatine and alisphenoid portions of the sphenoid ( Figure 1B, Figure 2). It serves as the origin for the M. pterygoideus medialis and the M. pterygoideus lateralis, upper and lower slips. The flanges are elongate, but not inflated in *Hapalops*, as in the tree sloth, *Bradypus* ( Naples, 1982).

**Temporal—**This element has small squamous and large zygomatic portions, with an anterior process forming the posterior zygomatic arch root. The anterior tip, medial and lateral margins of the process are sharp. A lateral triangular concavity renders the process wedge-shaped ( Figure 1B). A dorsal triangular concavity also serves as the origin of the most posteroventral fibers of the M. temporalis ( Figure 1A). It flares to the widest point at the widest part of the braincase. Ventral to the posterior zygomatic root merging with the braincase is the large, external auditory meatus, which has a large, rounded, laterally facing opening ventral to the posterior zygomatic root origin. The mastoid process is a broad descending projection with a pointed tip, near the posterior aspect of the skull.

### Character of cranial sutures in *Hapalops*

Most mammalian sutures are complex; however, in *Hapalops*, the interpremaxillary, premaxilla-nasal, and the nasal-frontal sutures are simple, abutting approximately linearly. Retaining these open sutures, even in aged adults, appears to be common in sloths. However, not all of the sutures in *Hapalops* are simple in structure. The most anterior suture showing the typical complexity is the frontoparietal. In one specimen (FMNH P13141), bilateral sutural bones occur between the frontals and parietals, further increasing sutural complexity in this area [( Herring, 1972; Herring, 1974; Jaslow, 1989; Jaslow, 1990; Herring & Mucci, 1991; Herring & Teng, 1995; Herring, 1997) Figure 1]. Other, especially more posterior sutures, become increasingly complex.

### Ventral view characters

**Figure 4.**
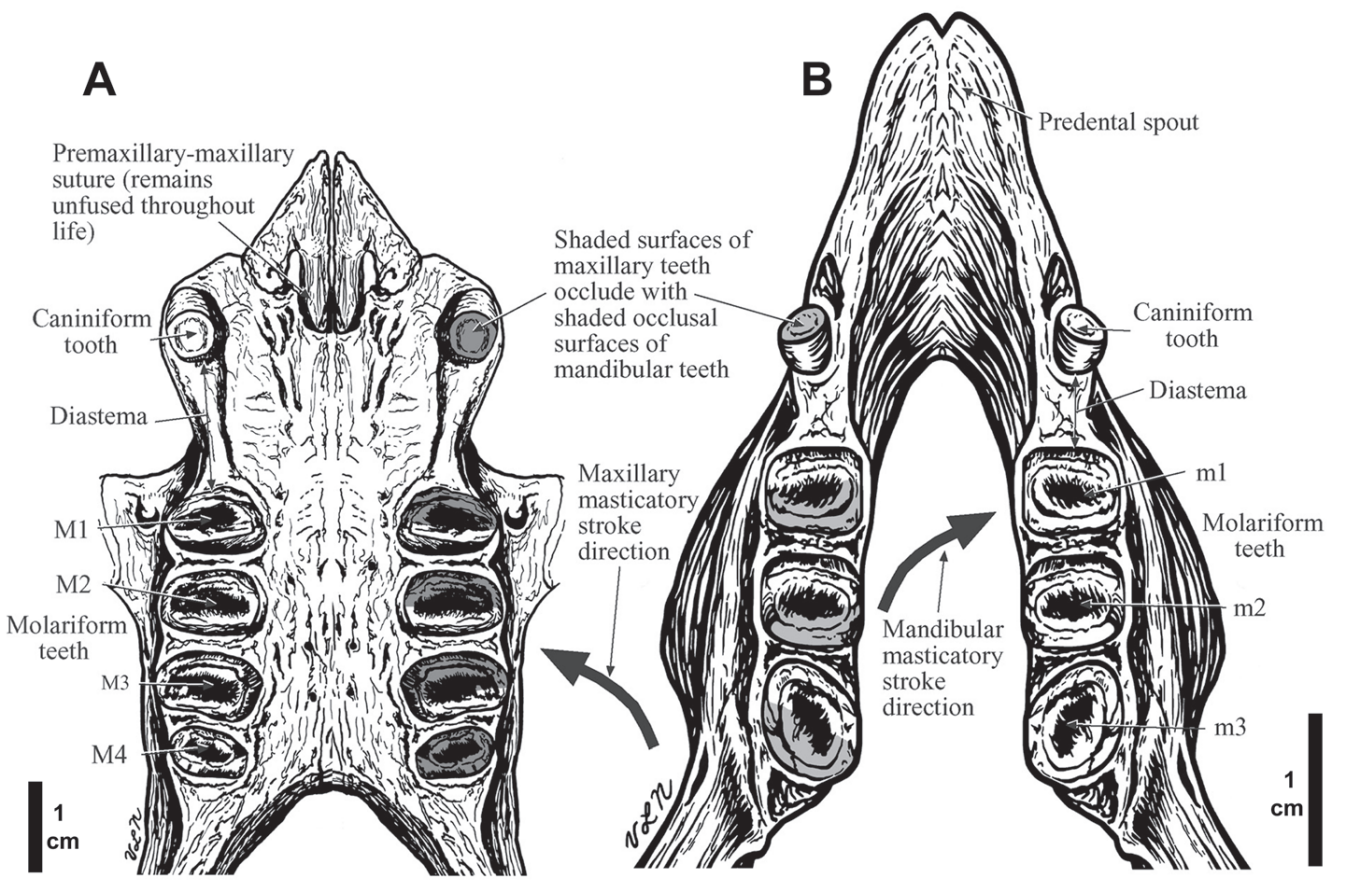
The maxillary ( **A**) and mandibular ( **B**) dentitions of *Hapalops* are shown. The shaded and unshaded surfaces of maxillary teeth occlude with corresponding surfaces of mandibular teeth. Arrows indicate the orientation of the masticatory power stroke which emphasizes the mediolateral component of mandibular movement.

**Palate—**The anterior premaxillary portion is pointed and narrow, widening greatly at the maxillary suture anterior to the caniniform tooth alveoli ( Figure 2, Figure 4A). Posterior to the caniniforms the palate constricts, widening again anterior to the molariform toothrow. The hard palate ends at the posterior margin of the last upper molariform. It is rugose, with many pits and nutrient foramina in a double row parallel to the midpalatal suture. In some specimens the medial suture is partially fused, and when so, it is complex. The palate narrows posteriorly, ending in a “C” shape with the open ends posterior at the choanae ( Figure 2). The origin scar of M. uvulae is a pair of elongate ridges anterior to the posterior margin of the bones that mark the anterior soft palate edge, which continues farther posteriorly. The pterygoid hamulus is a small tubercle for the anterior origin of M. constrictor superioris, which may continue posteriorly along the ventral margin of the pterygoid flanges, although the continuation of this attachment is indistinct. Space between the palatine and pterygoid flanges is wide for the length of the region. The maxillary-palatine suture is only sometimes discernible. Anterior to the ridges and medially, two elongate depresssions serve as the anterior origins of M. tensor veli palatini. In specimens with well-preserved interpterygoid regions, a pair of large depressions with anteroposteriorly oriented long axes occurs medial to the palatine-pterygoid suture. Posterior to these depressions is a second shallower pair. Farther posteriorly is a pair of raised rounded ridges that are the M. tensor veli palatini origins. A second pair of rugose oval depressions with anteroposterior long axes occurs posterior to the tensor veli palatini ridges where the M. levator veli palatini inserts.

**Temporal—**The glenoid fossae are smooth, ovate depressions with mediolateral longer axes, and without prominent pre- or postglenoid processes. Anterior to the external auditory meatus is the elongate insertion scar for the posterior slip of M. levator veli palatine ( Figure 2).

**Basioccipital—**On either side of the midline is the roughened raised basilar tubercle that continues posteriorly as a ridge ending immediately anterior to the raised lip of the anterior edge of foramen magnum ( Figure 2). This feature is the posterior origin of M. constrictor superioris. Posterolateral to the tubercles and anterior to the occipital condyles is an ovate depression for the M. longis capitis insertion. The foramen magnum is a large oval with a horizontal longer axis. It opens posteroventrally, and is most visible ventrally. There are no tympanic bullae. There is a prominent stylohyal pit, which serves as the stylohyoid bone articulation. The stylohyal pit is far posterior on the skull, anterior to the lateral aspects of the occipital condyles. The prominent mastoid processes are lateral to this feature.

### Occipital view characters

**Figure 5.**
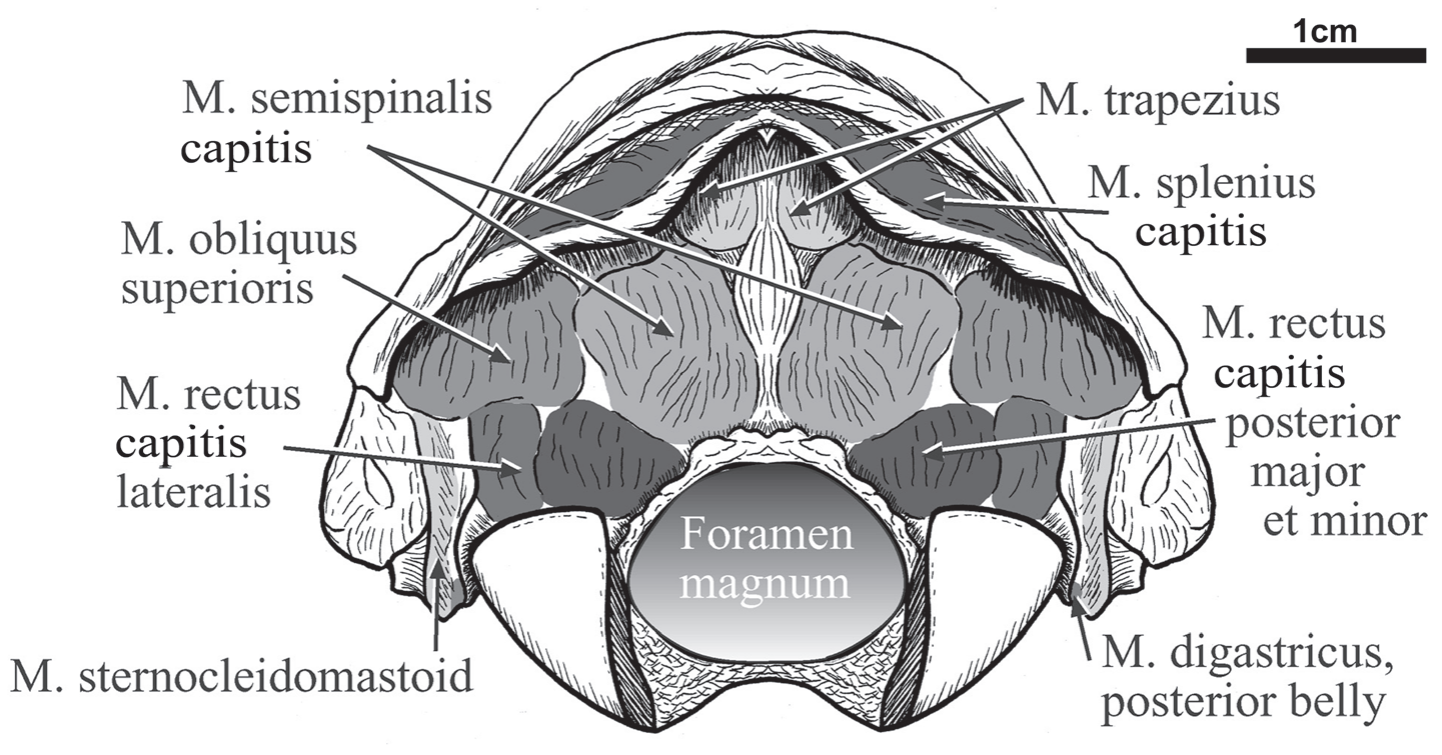
The skull of *Hapalops* is shown in occipital view. Scars of origin of the suboccipital muscles are indicted by shading.

The nuchal crest is prominent with deep, triangular scars for insertion of the M. trapezius; a broader, shallower depression is the insertion of the M. splenius capitis, and posteroventrally a sharp-edged process is the M. sternocleidomastoideus origin ( Figure 5). Ventral to the nuchal crest are roughened scars for insertion of the M. semispinalis capitis, M. rectus dorsalis posterior major *et* minor, and the M. obliquus capitis cranialis. Scars for the M. rectus capitis lateralis insertion occur lateral to the M. rectus dorsalis posterior major *et* minor insertions. The posteromedial aspect of the descending ridge, the M. sternocleidomastoideus origin, shows a small oval depression bounded by a roughened ridge; this is the origin of the M. digastricus posterior belly. The occipital condyles are large for the skull size, as is common in long headed sloths. They are widely spaced mediolaterally, with the rounded articular surfaces extending dorsally beyond the vertical, covering about 200 degrees. Condylar convexity in the horizontal plane allows about 150 degrees of mediolateral head movement.

### Character of sloth dentition: Maxillary

Sloths have ever-growing teeth. *Hapalops* has a dental formula reduced compared to other mammals, but with the maximum number of tooth types for sloths; anterior upper and lower caniniform and four upper and three lower molariform teeth ( Figure 4). The upper caniniforms are rounded, recurved triangles with a single flat wear facet facing posteriorly ( Figure 1B). Sloth teeth lack enamel, but have a harder outer and a softer inner dentine layer, with central tooth basins formed from wear ( Naples, 1987). The caniniforms occur at the widest part of the palate, with roots large enough to cause labial bulges in the maxilla. A diastema separates these teeth from the more posterior molariforms. The first molariform is a rounded rectangle, wider labiolingually. The harder outer dentine “shell” is thinner than that of the caniniform, and the central basin is approximately transverse; slightly more anterior lingually ( Naples, 1982; Naples, 1987; Naples, 1990). In our specimens, the harder outer dentine was worn through into the softer inner layer, as is true in all but the youngest sloths ( Naples, 1982; Naples, 1987). Flat wear surfaces angled anterodorsally and posterodorsally occur on both anterior and posterior faces of the first three molariforms. The second molariform is also a rounded rectangle, separated from the first by approximately 25% of the anteroposterior length of the first tooth, a spacing also shown between the more posterior molariforms. The second molariform is the largest, approximately rectangular, but with more rounded anterior and labial faces. The third molariform is a more rounded trapezoid than the first two, with the widest face anterior. In several specimens, the central basin is directly transverse rather than slightly anterior labially. The fourth molariform occlusal surface is half the anteroposterior diameter of the third, and about 80% as wide, with a single large anterodorsal wear facet and a smaller posterodorsal one. Even though the molariforms are different in size and labiolingual width, the interdental palatal width is equal anteroposteriorly. Occlusion in *Hapalops* ( Figure 4) resembles that in *Choloepus* ( Naples, 1982).

### The mandible: bony features, muscle scars and dentition

**Figure 6.**
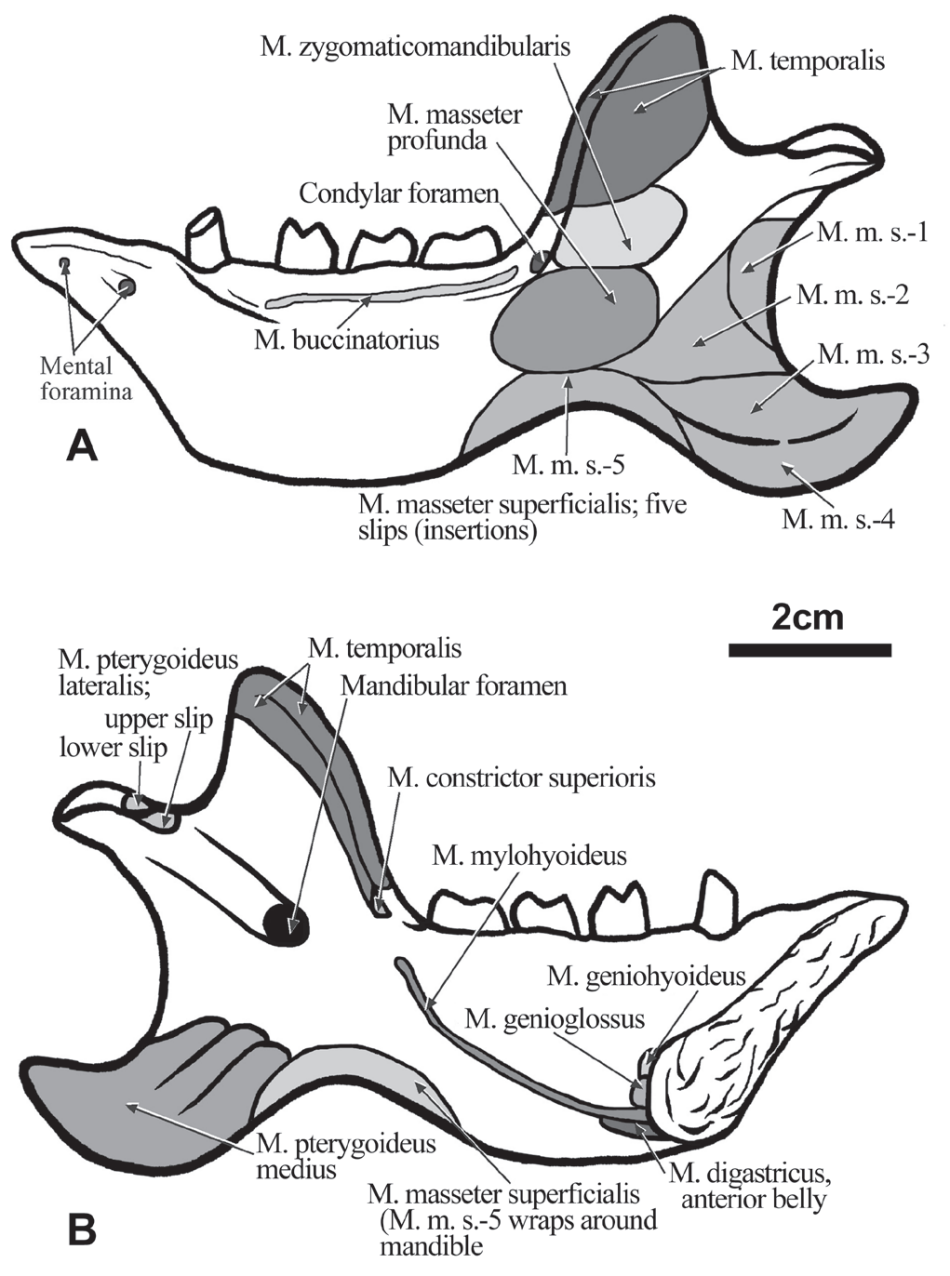
The mandible of *Hapalops* is shown in lateral view ( **A**) and lingual view ( **B**). The scars of muscle origin and insertion are labeled and shaded.

**Dorsal view characters—**The predental spout dorsal edge is sharp, and the outer surface rugose ( Figure 4B) with a smooth lingual “U-shaped” depression. The symphysis is broadly fused and anteroposteriorly deep, with no traces of the intermandibular suture. There is a shallow depression anterior to the lower caniniform, to accommodate the tip of the upper caniniform at full dental occlusion. It is a small, triangular-shaped area, with the apex directed anteriorly. There is a flattened rugose area between the caniniform tooth and the first molariform, *i.e*., the diastema. The dorsal surface of the bone surrounding the molariforms is slightly roughened and more porous than that on the labial or lingual aspects of the mandible. A depression dorsal to the M. buccinator attachment marks the gingival attachment, and parallels the toothrow lateral to the molariform alveoli ( Figure 6A). The alveoli are bounded labiolingually by sharp ridges that converge and continue posteriorly as a single, sharp-edged ridge, lingual to the coronoid process. The coronoid process is thicker anteriorly, and thins posteriorly. The mandibular condyles are ovoid, with a greater lingual flare and a gentler lingual slope.

**Lateral view characters—**In profile, the dorsal surface of the predental spout varies from rounded to a sharply pointed anterior tip ( Figure 6), and is elevated at an anteriorly directed angle from the dentition. Especially marked in specimens with rounded, blunt anterior predental spout profiles (AMNH 9222 and KU 145120), a shallow “V-shaped” notch occurs on the anterodorsal tip when the mandible is viewed anteriorly. Regardless of shape, a thickened bony border reinforces the dorsal rim ( Figure 4B). Ventral to the rim, the spout shows a slight concavity ( Figure 4B, Figure 6A), as seen in other sloths with caniniforms laterally displaced from the main axis of the tooth-row ( McAfee, 2007). The shallow depression for the upper caniniform is also visible labially. About halfway between the tip of the spout and the caniniform is a large mental foramen; a smaller, additional one sometimes occurs near the tip ( Figure 6A). These openings allow passage of one or more large mental nerve branches that innervate a large, flexible lower lip. The ridged, thick dorsal boundary of the spout continues posteriorly the length of the toothrow. Ventral to it is a deep depression between the caniniform and first molariform that ends in a ridge formed by the root of the posterior portion of the first molariform ( Figure 4B). This tooth is tilted at 30 degrees anterior to vertical. The posterior ridge border is bounded by a second, smaller depression. A second similarly inclined ridge occurs labial to the bulge of the second molariform, followed by a shallower depression anterior to the third tooth. The ridge labial to the third molariform is less pronounced, and more vertical than previous ones. Grooved ventral borders of the depressions mark the labial attachment of the oral gingiva, underlain by the M. buccinatorius ( Figure 6A). Posterior to the posterior end of this scar, at the base of the coronoid process, and posterior to the third tooth, is a large elongate and oval condylar foramen with a labially directed opening ( Figure 6A). This character is an autapomorphy for sloths, and among other mammals has only been observed in the platypus, *Ornithorynchus aculeatus* (personal observation). The coronoid process is large and broad anteroposteriorly, with a convex anterior edge and a rounded apex. The posterior face descends to a broad, shallow condylar notch. A ridge that continues dorsally along the labial surface begins dorsal to the condylar foramen, parallels, and then moves onto the leading edge. A second ridge runs dorsally, parallel to the more anterior one. A third ridge marks the posteroventral boundary of the M. temporalis insertion, from opposite the toothrow level to slightly dorsal to the coronoid notch ( Figure 6A).

The mandibular condyle is gently convex, with some articular surface visible labially ( Figure 6A). A labial ridge from the posterior edge buttresses the condyle, extending anteroventrally, fading labially. Depressions and rugosities, separated by ridges, mark labial masticatory muscle insertions. The angular process is large, convex ventrally, and projects posteriorly farther than the condyle. The dorsal aspect is ovate, with the long axis anterolateral to posteromedial. The articular surface with the glenoid fossa is convex, and “C-shaped” with the open arms facing anteriorly.

**Lingual view characters—**As in other sloths, the mental symphysis in *Hapalops* is fused ( Figure 4B). Nevertheless, fossil sloth mandibles are often represented by a single ramus, with the break at or near the symphysis, perhaps because despite its robustness, it is weaker than the mandibular body housing the large ever-growing molariforms. The symphysis is thick in cross-section, particularly ventrally, thinning dorsally, toward the anterior tip of the spout. The mandibular lingual surface is smooth, although marked by the mylohyoid line, the M. mylohyoideus origin from the posterior symphysis, level with the mental spine. It trends posterodorsally, ending posteriorly ventral to the tubercle where the anterior M. constrictor superioris originates. Posterior to the last molariform, this tubercle may continue anteroposteriorly as a ridge, projecting lingually at the base of the anterior part of the coronoid process ( Figure 6B). A third ridge marks the posteroventral boundary of the M. temporalis insertion from opposite the toothrow to slightly dorsal to the coronoid notch. The lingual coronoid surface bears anterior and posterior insertion scars for M. temporalis slips ( Figure 6B). The M. pterygoideus medius insertion is large, and occupies most of the angular process. Unusual in comparison to other sloths, in *Hapalops* this region is subdivided by two posteroventrally curving parallel ridges.

Posterior to the coronoid notch, the mandibular condyle is convex. The ventral articular surface, visible lingually, is sculpted by rugose, dorsal and ventral ovoid insertion scars with longer anteroposterior axes; the lower more elongate, for the M. pterygoideus lateralis. In some cases, the lower scar is oriented toward the groove that leads anteriorly to the mandibular foramen. This unusually large foramen is a sloth autapomorphy, and in *Hapalops* a broad, deeply incised groove leads to it, proceeding anteroventrally from the condylar notch dorsally, and the ventral border of the condylar process ventrally.

**Figure 7.**
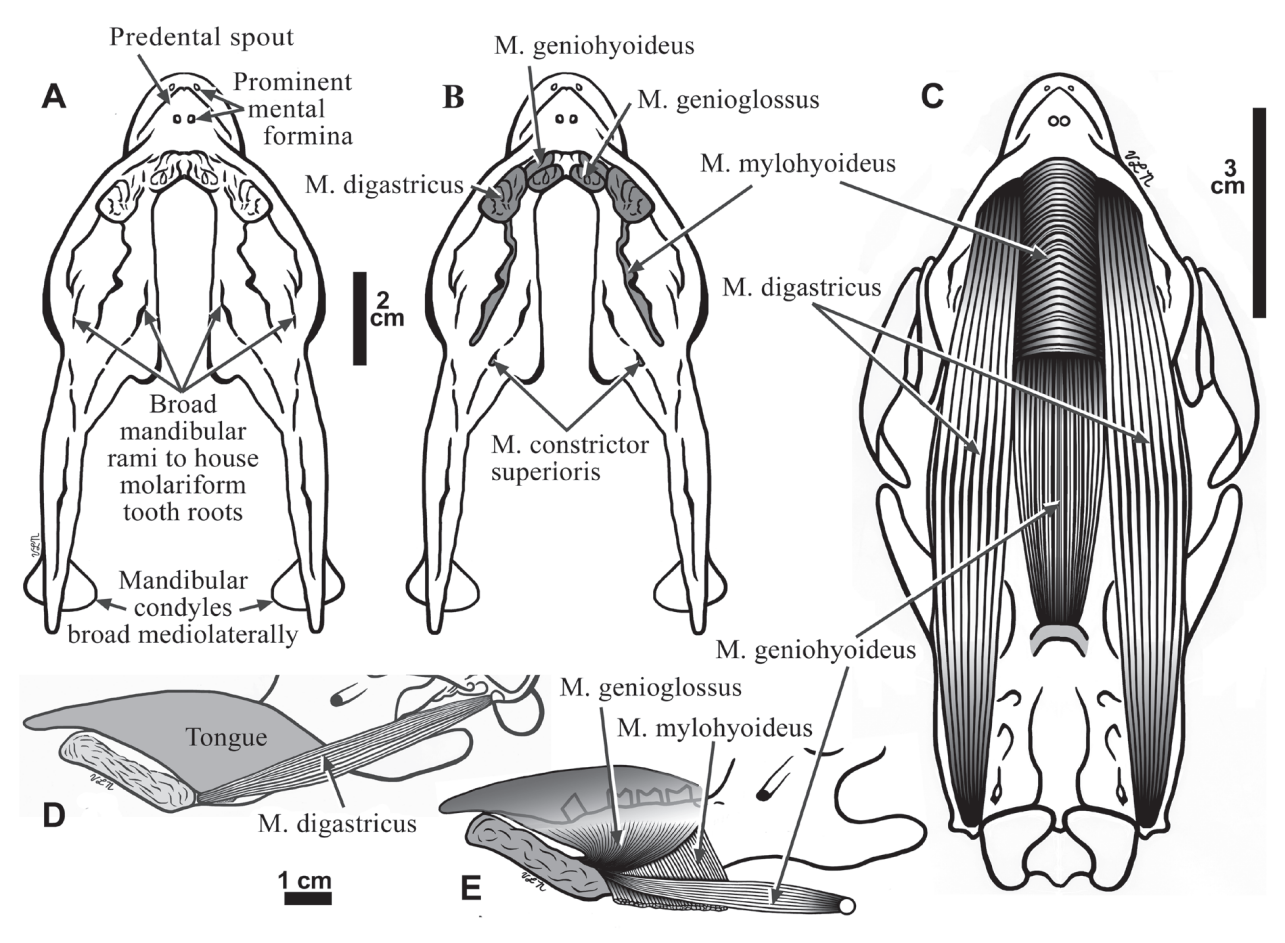
Ventral views of the mandible of *Hapalops* showing suprahyoid muscle scars ( **A**) and with areas of muscle origins shaded ( **B**); 2 cm scale. These muscles are reconstructed in ventral view ( **C**) and lateral view ( **D**, **E**). In **D** and **E**, the tongue position has been estimated to show it relative to that of the oral musculature; 1 cm scale.

**Ventral view characters—**The predental spoutis elongate, slender and pointed, with a sharp midventral ridge ( Figure 7A, 7B). The ventral mandibular surface is rugose with oval scars for the M. genioglossus origin on each side of mental spines at the midline. Posterior to this feature is a larger, oval, ridge-bounded rugosity that is the M. geniohyoideus origin. Posteriorly a much larger ridge-bounded roughened rectangle with rounded corners extends distally as far as the bulge housing the molariform roots in the mandibular body. This scar is for the M. digastricus, anterior belly insertion. It is prominent in *Hapalops*, but smaller than in the tree sloths *Bradypus* and *Choloepus* ( Naples, 1985a).

**Mandibular dentition—**In adult sloths tooth root diameters equal the erupted portions, and extend almost to the ventral aspect of the mandibular body ( Naples, 1982; Naples, 1990). They are surrounded by thick alveolar walls, and together cause labially and lingually rounded bulges in the mandibular body, similar to maxillary bulges for the upper caniniforms ( Figure 4B). The first lower molariform grows vertically, but the second and third are increasingly oblique, with the occlusal surfaces more lingual. The increasingly oblique orientations toward the posterior aspect of the toothrow, as well as their spacing form the wear-induced dental occlusal surface shapes [( Naples, 1985a) Figure 4]. Oblique tooth orientation allows the toothrow width to remain equal anteroposteriorly, while allowing increased space posteroventrally for housing the tongue base and other soft oropharyngeal tissues.

Each ramus has four teeth, an anterior caniniform and three posterior molariforms ( Figure 4B). The caniniforms are rounded triangles, with a single articular facet slanted anteroventrally, separated by a diastema and slightly laterally displaced from the longitudinal toothrow axis. The first and second molariforms are rectangular, with an anterior anterodorsally facing facet and a middle basin, bounded posteriorly by a posterodorsally facing facet. All molariforms are separated by large interdental spaces, an unusual feature in herbivores but common in sloths. The second molariform occlusal surface is slightly larger than the first. The third molariform surface is generally smaller, but more variable, ranging from diamond shaped, with the long axis oblique, labial side more anterior, to rectangular. In a few animals it is larger than the more anterior molariforms.

**Figure 8.**
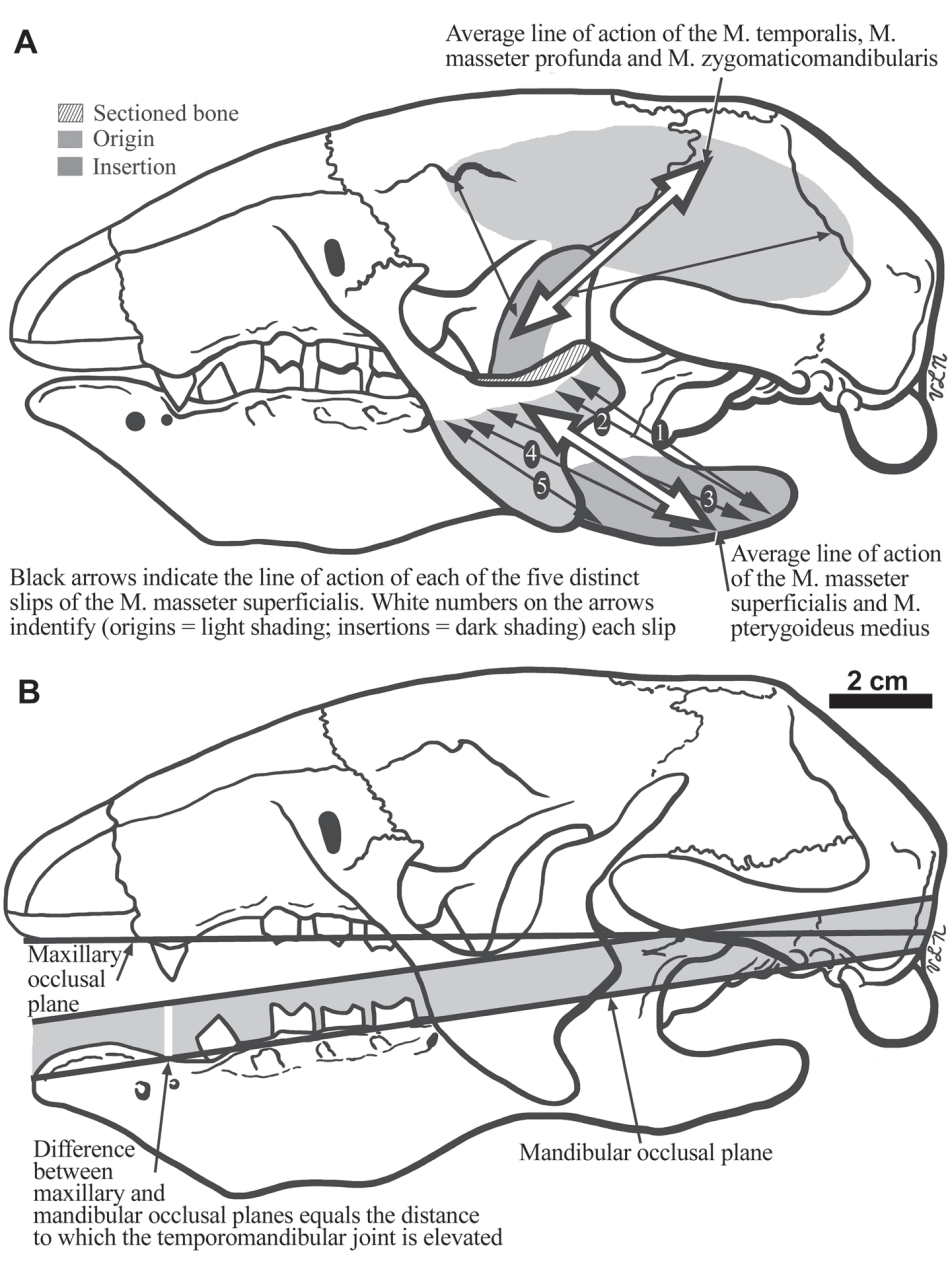
The cranium of *Hapalops* is shown in lateral view ( **B**), illustrating the differential height of the craniomandibular joint for the skull and mandible. In **A**, the lines of action of the five individual parts, and a composite line of action of the M. masseter superficialis, and the anterior and posterior as well as a composite line of action for the M. temporalis are illustrated.

**Tooth occlusion—**Occlusion in *Hapalops* is similar to the pattern in other sloths studied [( Naples, 1982; Naples, 1985; Naples, 1987; Naples, 1989) Figure 4]. Caniniforms occlude with the maxillary tooth anterior to the mandibular, each with a single facet. This is opposite to the canine occlusion pattern in other mammals; hence these teeth are unlikely canine homologues. Molariform tooth homologies are also unknown, as no sloth has a deciduous dentition to allow study of tooth eruption sequences. As with the caniniforms, the upper molariforms occlude half a tooth length anterior to the mandibular molariforms with the anterior faces of the maxillary tooth central basins occluding with the anterior faces of similarly numbered mandibular molariform teeth. The posterior sides of the central basins of the upper molariforms occlude with the anterior faces of the central basins of the corresponding lower molariform. The single anteriorly facing facet of the upper fourth molariform occludes against the posterior face of the lower third molariform. The tooth positions suggest that when the caniniforms are in occlusion, the molariforms are not, as is the case in tree sloths [( Naples, 1982: Figures 4C, 4D) Figures 4; 8].

### Reconstruction of cranial ligaments in *Hapalops*

Other than a few mammals with complete bony postorbital bars, such as primates and a few felids ( Romer, 1966; Naples, 1982; Naples & Martin, 2000a; Naples & Martin, 2000b; Kardong, 2005), or a postorbital wall, as in humans and derived barbourofelins ( Naples & Martin, 2000b; Standring *et al.*, 2005), the usual condition is a bony postorbital process partially separating the orbit from the temporal fossa. In sloths, as most other mammals, a ligament connects the posteroventrally projecting bony supraorbital process to a corresponding posterodorsally projecting edge or process of the jugal. In sloths a ligament between the anterior and posterior portions completes the zygomatic arch and serves in lieu of the complete bony arch ( Kardong, 2005). There is also a second ligament, the postorbital, as in most mammals, which connects the postorbital process to the jugal bone. The third set of cranial ligaments surrounds the craniomandibular joint, constraining the direction and degrees of freedom of movement of the mandibular condyle in the temporal glenoid fossa.

**Zygomatic arch ligament—**An unfused zygomatic arch is a derived character that distinguishes pilosans from most other mammals ( Naples, 1999), but is plesiomorphic within the group. The form varies among the living and extinct sloths; however, it is partly affected by overall cranial size and proportion. Observations from tree sloth dissections and reconstructions of medium-sized fossil sloths illustrate how skull proportions and zygomatic morphologies differ from *Hapalops* ( Naples, 1982; Naples, 1985a; Naples, 1987; Naples, 1989). Specifically, the ascending jugal process is more vertically oriented than that of all other sloths studied herein, except for *Bradypus* [( Naples, 1982) Figures 1B; 3A; 8B)]. However, this feature differs from *Bradypus* because the cranium in *Hapalops* is elongate, while tree sloth’s is the most anteroposteriorly shortened among known sloth lineages. Therefore, both the postorbital ligament and the zygomatic arch ligament in *Hapalops* are shallower dorsoventrally than in *Bradypus*; these positions allow a shorter vertical but anteroposteriorly broader area for attachment of the portions of the masseter and temporalis muscles arising from them. These differences in the origins and insertions of these muscle segments do not resemble those of *Bradypus*, despite similarities of the shape and orientation of the zygomatic arch. These dissimilarities determine some of the biomechanical differences between *Hapalops* and all other sloths for which these characters have been elucidated ( Naples, 1982; Naples, 1987; Naples, 1989).

**Figure 9.**
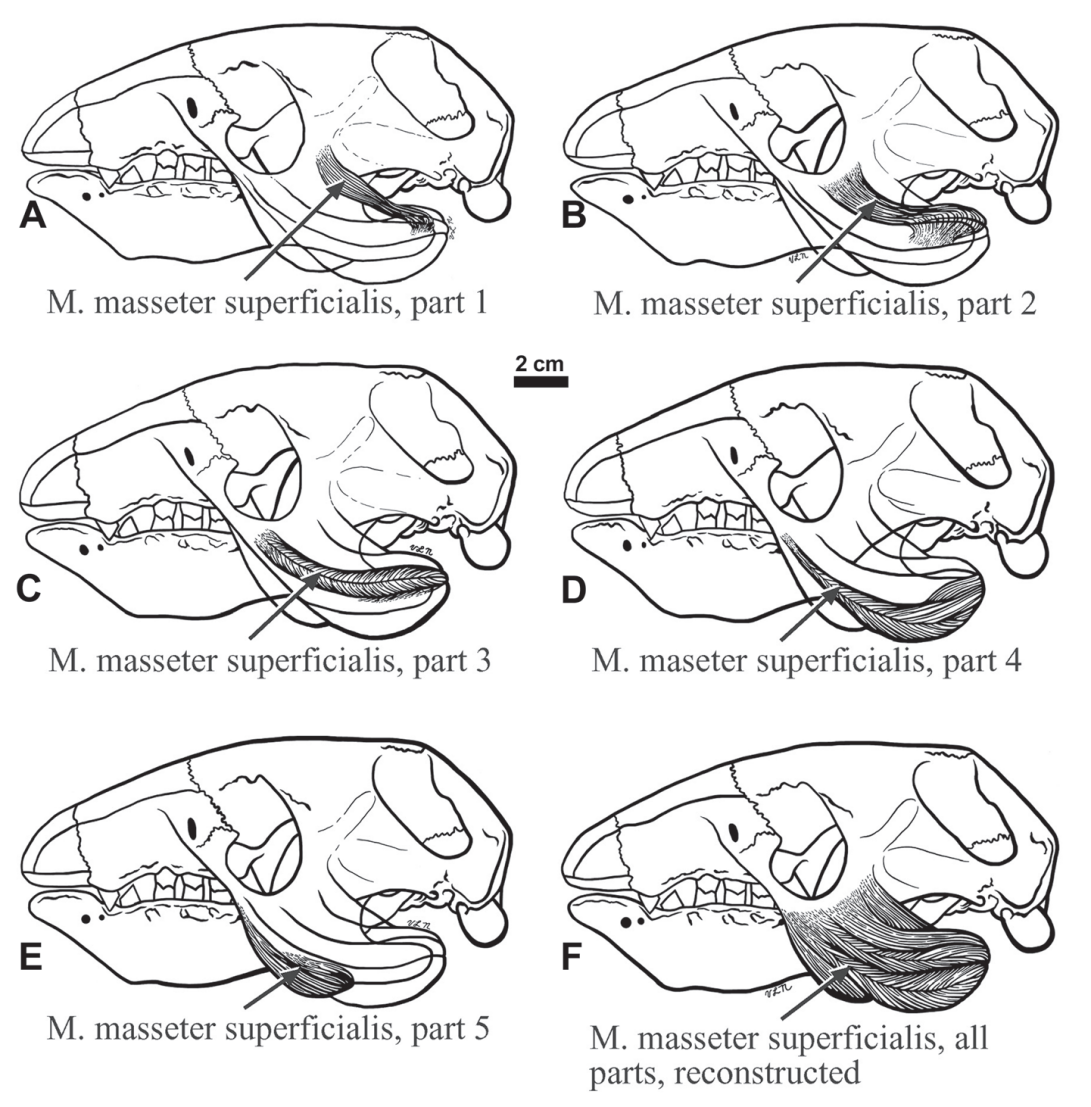
The five distinct parts of the superficial masseter muscle of *Hapalops* are shown in lateral view ( **A**– **E**), with a composite muscle restored in **F**. The muscle has been reconstructed based on the scars of origin and insertion, and in relation to those dissected in the tree sloths, *Bradypus* and *Choloepus*.

**Postorbital ligament—**In *Hapalops*, the postorbital process connects to the anterodorsal edge of the ascending process of the jugal by a ligament. This ligament forms the posterior limit of the orbit and overlies the anteriormost fibers of M. temporalis ( Figure 9). Ligament calcification occurs progressively as sloths age, causing the postorbital process of the frontal to lengthen and increase in robustness at the base ( Naples, 1982; Naples, 1985a; Naples, 1985b; McAfee, 2007).

**Craniomandibular joint ligaments—**These ligaments in *Hapalops* can be inferred from rugosities surrounding the glenoid fossa and condylar process neck, and by comparing these features to dissections of *Bradypus* and *Choloepus*. In both tree sloths, the joint is surrounded by a continuous sheet of fibrous connective tissue, interrupted only at the anteromedial “corner” by the tendons of origin of the two portions of the lateral pterygoid muscle.

### Reconstruction of cranial musculature in *Hapalops*

Previous studies ( Naples, 1985a) discussed tree sloth cranial musculature, and the details of their anatomy can be used to infer the arrangement of the same muscle groups in *Hapalops*. Muscle-bone attachments leave characteristic marks, such as rugose surfaces bounded by raised ridges. Divisions of individual muscles are also marked by elevated ridges, allowing estimation of the relative surface area of attachment of different muscle segments, as well as of entire muscles. These features are more prominent as animals increase in size, and *Bradypus* and *Choloepus* show the typical masticatory muscle pattern, including the same innervation (Trigeminal nerve, V _3_) as in other mammals ( Naples, 1985b). The musculature of the tongue, throat and hyoid regions also conforms to the typical mammalian pattern of divisions and innervations, such as discussed in: Sisson & Grossman, 1953; Crouch, 1969; Evans, 1993; Williams *et al.*, 1989; Anderson & Anderson, 1994; De Iuliis & Pulera, 2007; Schuenke *et al.*, 2007. Nerve branches in *Hapalops* could not be traced; however, the positions of cranial foramina are similar to those of the tree sloths.

### Masticatory musculature

**The masseter musculature—**The masseter muscle in living sloths is complex and uniquely subdivided into five superficial portions, or parts, and a single deep portion, separated by tendons and fascial sheets ( Naples, 1985a). Each of these divisions is easily discerned because it arises from a clearly marked region of the ventral process and posterior base of the dorsal process of the jugal and the ligament that connects the jugal and squamosal. A similar arrangement occurred in *Hapalops* ( Figure 3, Figure 8, Figure 9), although the relative positions and orientations of muscle parts in this sloth differ from those in the tree sloths.

**M. masseter superficialis, part 1—**This muscle arose from a rectangular depression with rounded edges, oriented approximately 45 degrees posterior to vertical. The fibers trend posteroventrally to insert into a crescent-shaped, lateral depression of the mandibular coronoid process. This superficial masseter muscle segment insertion is the most posterodorsal.

**M. masseter superficialis, part 2—**This segment originates from a “U-shaped” depression that covers most of the lateral root surface of the ascending jugal process. The muscle inserts into a triangular depression anterior and slightly ventral to that of part 1, and has a similar orientation.

**M. masseter superficialis, part 3—**This segment’s origin encompasses the largest elongated oval depression on the lateral surface of the descending jugal process, from an elongate depression ventral to the horizontal ridge thickening the transverse portion of the jugal passing ventral to the orbit. This muscle’s insertion is from the posterior mandibular body edge to the angular process tip, immediately ventral to the part 2insertion, and shows a line of action similar to parts 1 and 2.

**M. masseter superficialis, part 4—**This portion arises anterior to part 3, continuing distally along the descending jugal process to the tip, which it encompasses. The insertion occupies a crescent-shaped depression immediately ventral to the ridge of attachment for the most ventral tendon bounding part 3, continuing along the ventral mandibular margin. This segment’s line of action is more horizontal than the muscle average.

**Figure 10.**
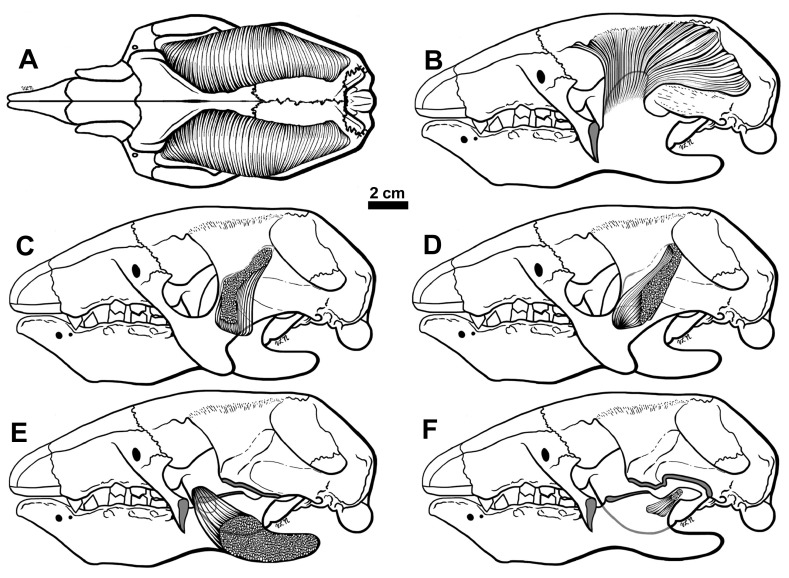
Reconstructions of the M. temporalis in dorsal view ( **A**) and lateral view ( **B**), the M. masseter profunda ( **C**), M. zygomaticomandibularis ( **D**), M. pterygoideus medius ( **E**) and M. pterygoideus lateralis ( **F**). In **B** the mandibular coronoid process is represented as if the muscle inserting on it was transparent. In **C** and **D**, the zygomatic arch and cranial ligaments are shown as if transparent. In **E**, both the anterior and posterior portions of the zygomatic arch are cut (indicated by shading), as well as the ascending portion of the coronoid process. The mandible is depicted as transparent to show the insertion of the muscle on the medial side. In **F** the portions of the zygomatic arch are cut as in **E**, and the shape of the pterygoids flanges are shaded grey. The mandible is also depicted as transparent, with the insertion of the M. pterygoideus lateralis shows attaching to the medial side of the condylar process. This muscle has been restored with two heads as is the case in tree sloths.

**M. masseter superficialis, part 5—**The dorsal part of the origin is anterior to parts 3 and 4, continuing distally from an elongate, narrow ovate depression paralleling the anterior descending jugal process edge. The insertion is into a crescentic depression that echoes the concavity between the mandibular body and the angular process, wrapping around the ventral edge. The relative size, orientation and position of these attachments determine the degree to which the mandible can open because this segment is stretched to the greatest extent upon mandibular depression. The line of action of this segment is the most horizontal. Together, the five M. masseter superficialis segments show a wide range of individual lines of action, therefore functioning at different parts of the total range; their average also shows the overall muscle orientation ( Figure 10B), which is more horizontal in *Hapalops* than *Bradypus*.

**M. masseter profundus—**In *Hapalops*, the origin is from an elongate oval depression on the medial aspect of the lower two-thirds of the ascending portion of the jugal and the proximal half of the descending process. It inserts into an oval depression with a longer anteroposterior axis on the mandibular body dorsal to M. masseter superficialis, part 5 ( Figure 3B, Figure 10C).

### Temporalis musculature

In *Hapalops* the muscle arises from an anteroposteriorly elongated oval fossa on the lateral braincase surface (squamosal and parietal) and the posterior dorsolateral walls of the frontal. The muscle does not meet at the dorsal midline, as is true of other sloths that have been studied; the superior temporal line is clearly marked on the lateral sides of the braincase ( Figure 1). It is not possible to determine whether the origin was divided into superficial and deep portions; however, because of the shape of the temporal fossa it is a relatively large muscle. The insertion covers muscle scars on the anterior, lateral and medial portions of the large, prominent coronoid process ( Figure 6, Figure 9, Figure 10B). Although the average muscle orientation trends anteroventrally, the great anteroposterior temporal fossa length indicates that there is a range of lines of action, from nearly vertical anteriorly to almost horizontal posteriorly ( Figure 9A).

**M. zygomaticomandibularis (= M. temporalis, deep portion)—**This muscle arises on the deep zygomatic arch face and the ligament uniting it, from an ovate scar which is broader dorsally than ventrally ( Figure 3B, Figure 6A, Figure 10D).

### Pterygoideus musculature

These muscles are large relative to the pterygoid mass in other mammals, although they show the same pattern of division ( Toldt, 1905; Toldt, 1907; Toldt, 1908; Edgeworth, 1935; Turnbull, 1970; Naples, 1985a).

**M. pterygoideus medius—**This muscle is relatively large and arises from an oval depression on the ventrolateral pterygoid flange ( Figure 1B, Figure 6B, Figure 10E). The muscle inserts on the rugose medial surface of the mandibular ramus and the large, posteriorly extended angular process.

**M. pterygoideus lateralis—**This musclehas dorsal and ventral heads, arising from ovate depressions anterolateral on the pterygoid flange. The upper head originates anterodorsal to the lower. These muscles insert separately onto the medial mandibular surfaces, anteroventral to the mandibular condyle ( Naples, 1982). The interruption of the joint capsule, the site of the insertions of the two tendons in *Hapalops*, is marked by a pair of adjacent depressions ( Figure 6B). In the tree sloth *Choloepus*, orientation of the M. pterygoideus lateralis heads is posteroventral, as in other mammals, but in *Bradypus* the heads are posterodorsally oriented ( Naples, 1985a). This arrangement is similar in *Hapalops* ( Figure 1B, Figure 6B, Figure 10F).

### Suprahyoid muscles

**M. digastricus—**In most mammals, this muscle has two bellies; the anterior has a small, ventral, fleshy origin from the mandible, and the posterior arises from the medial aspect of the mastoid process. The two bellies are united by a thick, short tendon. The bellies have different innervations (Mandibular Nerve V _3_ for the anterior and Facial Nerve VII for the posterior), confirming that they develop from different branchial arches. The anterior belly is often more robust, and in tree sloths it expands greatly, with a less robust posterior belly ( Naples, 1986). In *Hapalops*, the origin scar of the anterior belly is larger and more robust than in most other mammals, occupying a rectangular area on the ventral mandible. The posterior belly origin is from a small tubercle medially on the mastoid process ( Figure 5, Figure 7C, 7D), and from a thick tendon.

**M. stylohyoideus—**This muscle is absent in the tree sloths ( Naples, 1986), as is likewise for *Hapalops*. In the sloths studied, a stylohyal pit indicates that the stylohyal bone articulated directly into this basicranial depression on the anteromedial opening for the external auditory meatus ( Naples, 1982).

**M. mylohyoideus—**In most mammals, this muscle forms the oral floor, meeting at a ventral midline raphe. In *Hapalops* this muscle originated from a prominent lingual ridge of the mandibular ramus. The mandible is deep at the mental symphysis, and the anterior scar limit is near the ventral symphyseal surface. The mylohyoid ridge angles posterodorsally along the lingual mandibular body, continuing anteroventrally to the bony prominence of the posterior terminus of the labial ridge where it is joined by the ascending medial ridge on the condylar process ( Figure 6B, Figure 7B, 7C, 7E). No *Hapalops* hyoid elements were available for study; therefore the extent of the attachment of this muscle to the basihyal was not possible to determine, and it is assumed that the muscle was united by a midline raphe as in other mammals ( Naples, 1986).

**M. geniohyoideus—**In most mammals, this muscle arises from the mandibular midline dorsal to the mylohyoideus and inserts on the basihyal. It protracts the hyoid with a fixed mandibular symphysis, or assists with mandibular opening if the hyoid is fixed. In *Hapalops*, it originates from a robust, oval scar with a wider mesiodistal axis, lateral to the ventral aspect of the mental symphysis and posterior to the M. genioglossus scar ( Figure 6B, Figure 7B, 7E).

### Buccolabial musculature

**M. buccinatorius—**This muscle shows many patterns among mammals; complex, multilayered or simple. In tree sloths, it is simple, single-layered, with dorsoventral fibers from the maxilla labially to the mandible ( Naples, 1986). In *Hapalops*, a depression marks the labial maxillary surface anterior to the caniniform, continuing dorsally and then ventrally to pass posteriorly between the ventral aspect of the molariform roots. The muscle scar continues posterior to M4, then trends ventrally toward the maxillary-palatal surface ( Figure 1B, Figure 6A). The anterior mandibular attachment begins anterior to the lower caniniform and continues posteriorly to a short distance posterior to m3. The anteroposterior extent of origin and insertion scars suggest *Hapalops* had a long oral cavity, as is corroborated by other features ( Figure 2B, Figure 6A).

### Suboccipital musculature

In most mammals this muscle group consists of four muscles that primarily extend the head at the atlanto-occipital joint (M. rectus capitis dorsalis major *et* minor), and rotate the head at the atlanto-axial joint (M. obliquus capitis cranialis and caudalis). These muscle scars in *Hapalops* seem relatively larger than in other mammals, and occupy most of the rugose surface of the posterior occiput ( Figure 5).

### Extrinsic lingual musculature

Muscles of this group originate outside the tongue and insert within it. They control tongue position with respect to the mandible and hyoid apparatus. Of the two muscles of this group, M. styloglossus was unable to be reconstructed.

**M. genioglossus—**This muscle arises from an oval scar with the main axis mesiodistal, anterior to the larger M. geniohyoideus origin scar ( Figure 6B, Figure 7B, 7E).

### Laryngopharyngeal musculature

This muscle group supports the hyoid bones and larynx, and forms the pharyngeal tube and esophagus. Muscle scars for M. stylopharyngeus were not discernable for reconstruction.

**M. constrictor superioris—**In most mammals, this muscle is quadrilateral, originating from the pterygoid hamulus and plate, the pterygomandibular raphe, the posterior end of the mylohyoid line and by an aponeurosis to the pharyngeal basioccipital tubercle. The muscle is a sphincter and provides peristaltic contractions for swallowing. In *Hapalops* a prominent tubercle for the anterior origin occurs at the posterior end of the mylohyoid line ( Figure 6B). A pterygoid flange attachment is also possible, but damage to the bone surface in this area in all available specimens precluded observation. Nevertheless, the pterygoid flange shape eliminates the space for either a pterygoid hamulus or pterygomandibular raphe. The posterior origin for this muscle can be restored in *Hapalops*, however, from paired, rounded basioccipital tubercles ( Figure 2).

### Palatine musculature

This muscle group controls the soft palate position and facilitates. Only muscles for which origin or insertion scars were discernable are discussed. The unrestorable muscles include: M. palatoglossus, M. palatopharyngeus, and M. salpingopharyngeus.

**M. tensor veli palatine—**In most mammals, this muscle is a sheet originating from the sphenoid bone horizontal plate, posterior to the curved palatine crest and on the anterior aspect of the lateral pterygoid plate ( Figure 2). The insertion is into the uvula base, and acts to move the uvula laterally to the same side, or to tense the soft palate when both sides contract simultaneously. In *Hapalops*, a depression marks the anterior palatal attachment posterior to a distinct palatine crest; anterior to the crescent-shaped depression that is the M. uvulae origin. The posterior origin is from the lateral surface of the anterior portion of the pterygoid flanges posterodorsal to the M. pterygoideus medius origin. As in other mammals, this muscle was a thin sheet trending anteroventrally from the posterior origin, joining with fibers from the palatal origin, to insert into the soft palate and the uvula laterally. The scars marking the origins of the muscle in *Hapalops* are large, suggesting that the soft palate was elongate anteroposteriorly.

**M. levator veli palatine—**In most mammals, this muscle origin surrounds the carotid canal on the medial, anterior, and lateral surfaces as a crescentric temporal bone scar. It inserts into the soft palate laterally on each side, elevating this structure. In *Hapalops*, the carotid canal is large, round, and has a large triangular, anteromedial scar ( Figure 2). This suggests the muscle originating from it was also large, and projected anteroventrally to merge with the soft palate.

**M. uvulae—**In most mammals, this muscle forms the substance of the uvula, the soft tissue projection arising from the posterior midline of the hard palate and traversing the soft palate posteriorly as a plug to complete the oral seal prior to swallowing. The attachment in *Hapalops* is typical of other mammals, although particularly large ( Figure 2).

## Discussion

The characters reconstructed suggest that *Hapalops* was a selective feeding herbivore. The initial movement of the masticatory cycle would be determined by combined masseter and temporalis muscle actions in elevating the mandible until dental occlusion occurred. Once the dentition engaged, the mandible would have completed the stroke mediolaterally. This masticatory cycle is supported by the mediolaterally elongated glenoid fossa and the wear facets of the teeth. It would have been achieved by alternating contractions between the right and left components of the medial and lateral pterygoid muscles. Thus, while the initial movement of the masticatory cycle could produce a degree of crushing force, the greater amount of food processing could be achieved by the secondary half of the cycle provided by the grinding forces. A preliminary and unpublished assessment of bite force ratios for a few small-bodied sloths by one of the authors (RKM) showed the moment arm for M. masseter to have the greatest effect upon mandibular elevation. The greatest ratios for relative bite force were for the posterior dentition, which is common for many herbivores, and indicates a large input of initial force that could be then applied to the mediolaterally oriented grinding stroke.

Divergence of pilosans into anteater and sloth lineages likely occurred in the early Eocene of South America ( Delsuc *et al.*, 2004). Eocene habitat and geography suggest that pilosan ancestors were small, arboreal insectivores living in forest canopies ( Pascual & Ortiz Jaureguizar, 1990; Marshall & Sempere, 1993). Sloths originated from these ancestors by shifting their ecological niche toward herbivory in conjuction with climatic changes that created opportunities for arboreal and terrestrial browsers. The fossil record, in contrast to the molecular data, that links sloths to anteaters is poor, although sloths show many structural influences retained from myrmecophagous antecedents, such as elongate, tubular heads, small oral openings, long tongues, an anteroposteriorly lengthened oral cavity, unfused zygomatic arch, and large occipital condyles extending posterior to the skull that allow great cranial freedom of movement in all directions.

A challenge facing early megatheroiids would have been food processing, as adaptations for insectivory select for reduced masticatory musculature and dentition, exemplified in early anteaters. An adaptive shift toward herbivory for such animals would be complicated by reduced muscle origins and insertions from the incomplete zygomatic arch. This condition occurs in only a few other terrestrial mammal orders, such as pholidotes and insectivores ( Mivart, 1871; Parker, 1885; Nowak, 1999), and the extinct multituberculates ( Carroll, 1988); none of which are herbivorous. The remaining dentition is reduced relative to that of buccinator-pilosan ancestors in the simple, enameless dentine structure that is plesiomorphic for xenarthrans. The transitional protosloth ancestor is currently unknown, but the adaptive changes necessary to fill an herbivore niche had been achieved by the time *Hapalops* and all other sloth families first appeared in the Miocene fossil record of South America. While many conditions in *Hapalops* are derived beyond those in a hypothetical protosloth, changes relative to increasing masticatory efficiency that differ from anteater lineages can be examined.

Sloth features allowing herbivory include elaboration and elongation of the jugal processes and bridging of the incomplete zygomatic arch. The increased length, dorsoventral and anteroposterior orientations of these flanges initiate masticatory efficiency improvement by increasing surface area for support of a five-part superficial masseter, relatively larger and reoriented deep masseter and zygomaticomandibularis muscles. Correlated changes include ventral pterygoid flange extension for pterygoideus muscle insertion and increased temporal fossa length and depth for temporalis origin. In the mandible, mental symphysis fusion and anterior elongation to form a predental spout as a channel for protrusion of the long, slender tongue was retained from earlier forms. A deep mandibular body provided additional space for ever-growing tooth roots and modification of mandibular condylar elevation relative to the mandibular toothrow affected gape and extent of the oral opening.

In addition to taxonomic relationships, characters in *Hapalops* provide specific insights concerning potential functional behaviors. Cranial anatomy can be used to infer locomotor capabilities and feeding strategies, which supports the phylogenetic characters associated with the transition toward becoming a unique herbivore, as well as those developed by later megatheroiids. *Hapalops* has been proposed as able to engage in arboreal or semiarboreal locomotor postures ( Scott, 1903; White, 1993), both of which require different structural and functional capabilities than terrestrial postures. Although it should be possible to identify structures correlated with habitat usage and locomotor patterns, the most important and probably earliest adaptations that would allow a change of feeding ecology to herbivory, would occur in the cranial and cervical regions. These changes would be reflected in the position and shapes of cranial bones and soft tissues of the laryngeal and pharyngeal regions; including structures involved in feeding, deglutition and respiration ( Reidenberg & Laitman, 1991). Change in feeding habits in *Hapalops* from ancestral forms likely resulted from evolution of new or different feeding strategies. These correlate with head and neck movement capabilities, making the anatomy of these regions appropriate indices for denoting change in habitus. Arboreal or semiarboreal animals must orient the vertebral column vertically or nearly so to climb trees, requiring a head and neck that can be held at up to ninety degrees to the body.

**Osteological adaptations and correlations—**In *Hapalops*, cranial changes for masticatory muscles in jugal proceses, pterygoid flanges and the temporal fossa produced lines of action directing the masticatory stoke labiolingually. Masticatory muscle origins and insertions differed in later sloths, but the labiolingual masticatory stroke orientation was retained. Further changes among sloth lineages in masticatory muscle orientation are variations on the theme of increasing the efficiency of this novel approach to herbivory. The typical mammalian herbivore has a complete zygomatic arch-musculoskeletal complex, whereas sloths adapted an incomplete zygomatic arch into a unique functional complex by regaining the ligament between anterior and posterior portions ( Figure 3A). This ligament also serves as the origin, permitting expansion of deep masseter and zygomaticomandibularis muscles in *Hapalops* ( Figure 3). In later, larger sloths a bony connection forms, but remains unfused in all but the largest megatheriids, because the trend toward connection is related to allometric pressures for increased body size ( Naples, 1985a; Naples, 1987; Naples, 1989). Smaller, later ground sloths and tree sloths retain separate arch portions.

The anterior sutures between the premaxillae, nasals, frontals, maxillae, and zygomatic bones in *Hapalops* are simple, and the anterior root of the zygomatic arch never fuses, even in the largest, most rugose and robust specimens. Because sutures between the more posterior skull bones show the typical complex interlocking mammalian pattern, simple anterior sutures indicate greater flexibility in this region, possibly an example of cranial kinesis; a rare phenomenon in mammals. Bones that abut along a straight line allow more “give” than do entirely immobile interdigitating sutures ( Badoux, 1966; Buckland-Wright, 1972; Buckland-Wright, 1978; Herring, 1972; Herring, 1974; Herring, 1997; Herring & Mucci, 1991; Herring & Teng, 1995). The presence of simple sutures may be important in distributing twisting forces generated on the elongate anterior face by facial muscles, and contraction of the masseter musculature pulling on the anterior portion of the zygomatic arch ( Thomason, 1991).

Mandibular structure in *Hapalops* and other megatheroids is derived when compared to the anatomy of other sloth families and a hypothesized protosloth. These differences include a mandibular condyle elevated above the occlusal plane and a deep ventral bowing of the mandibular body. In contrast, the mandibular condyle level is more even with the mandibular toothrow in mylodontids, and slightly to greatly elevated in megalonychids. Ventral bowing of the mandibular body also occurs in non-megatheroid sloths, but to a lesser degree. These differences, along with the abundant variation in predental spout shape and length, suggest that the mandible was less prone to phylogenetic influences from myrmecophagous ancestors.

In spite of their obligate herbivory, the dentition in all sloths is reduced in tooth number and type, as is true in *Hapalops*. The anterior caniniform teeth are small and project no farther from the occlusal plane than the molariforms. As with other sloths, the relative size, shape, orientation and spacing of the cheek teeth in conjunction with the anteromedially directed masticatory stroke determine the shape of the occlusal wear surfaces.

In many mammals, such as *Choleopus* and carnviorans, elongate teeth serve as occlusal guides because once the tips engage, mandibular movement is restricted to the direction allowed by dental overlap. Additionally, elongate canine teeth that require precise piercing point alignment are constrained to a path of motion that emphasizes movement in the vertical plane. Such an occlusal path requires the craniomandibular joint level to equal that of the maxillary-mandibular plane ( Greaves, 1973; Greaves, 1974; Greaves, 1980; Naples, 1982; Naples, 1985a), but does not allow an increased mechanical advantage for the superficial masseter, the main masticatory muscle of herbivores ( Turnbull, 1970; Greaves, 1980). However, the caniniforms in *Hapalops* are short and have minimal interlock. As such, they are occlusal guides for only a small percentage of the masticatory stroke, allowing greater flexibility in masticatory muscle arrangement, and are unencumbered by the elevated mandibular condyle.

The hard palate in *Hapalops* is elongated and of constant anteroposterior width, beginning at the pointed anterior premaxillary apex and continuing posteriorly with the maxilla and palatines. The marginal ridge on the posterior edge of the palate and reduced nasal spine are likely origins for M. uvulae and M. tensor veli uccinat. Together, these muscles elevate the uvula and tense the soft palate, sealing the oral cavity during food intake and mastication, and assist in pharyngeal opening during initiation of swallowing. The large origins of these muscles suggest they were of greater importance in *Hapalops* because of the great anteroposterior length of the oral cavity and posterior displacement of the pharyngeal entrance. Further evidence of an elongated oral cavity in *Hapalops* is demonstrated by the lengthy region between the pterygoid flanges and the paired ovate depressions marking the posterior origins of the superior pharyngeal constrictor muscles. The posterior origin is from the basilar tubercles anterior to foramen magnum; these tubercles in *Hapalops* and other sloths are apomorphies as this feature is usually single in mammals. Together, these attachments anchor the pharyngeal tube musculature, demonstrating it to be anteroposteriorly lengthened. This morphology indicates there was sufficient space for a long tongue with a great ability to protrude and retract. Elongation of this part of the muscular tube leading to the esophageal and glottal openings also suggests that *Hapalops* had great freedom of movement to elevate and depress the head; movements helpful for feeding and alteration of body orientation ( Reeve, 1940; Naples, 1999).

The scar for gingival attachment in *Hapalops* is more ventral on the labial side of the mandible than in many other animals. This indicates presence of a large buccal pocket labial to the molariforms, allowing the sloth large, flexible cheeks. This permits a large buccal cavity for manipulation of vegetation, as well as sufficient space to house a large, elongate tongue.

 The narrow palate and pointed predental spout suggest *Hapalops* was a selective feeder, as opposed to a bulk or grazing feeder as are herbivores with wide muzzles. The anteriorly pointed premaxillae further suggest a nipping/cropping action as the most likely method for food acquisition. The delicate nature of the premaxillae reduces the likelihood of *Hapalops* having strong, prehensile lips, but the rugose predental spout could have served as a sort of cutting board for vegetation. Most sloths need a cropping mechanism, as a result of the far posterior placement of the dentition. However, unpublished data determining relative bite force by one of the authors (RKM) indicates that the amount of force generated during the initial masticatory stroke in *Hapalops* to be much weaker than that of the similarly sized buccinators, *Nematherium*. With the lack of strong, prehensile lips, this places a greater emphasis on the need for *Hapalops* to engage in selective feeding, possibly on softer foods. The oblique wear facets between the caniniforms, along with the mandibular depression anterior to the lower caniniforms for the upper caniniforms could indicate an ability to shear more difficult foods if ingested laterally, and could constitute an additional means for cutting food. Shortness of the hard palate posterior to the last upper molariforms also contradicts a bulk feeding strategy. Animals ingesting a larger bolus need an increased palatal region for crushing food before swallowing. *Hapalops*, with a narrow palate, could only process small food boluses intraorally, although the capacious buccal cavity would have permitted shifting of food transversely during repetitive mediolateral chewing cycles. All of these factors contribute to this animal being a selective browser.

Many mammals have a styloid process, an elongated bony spur, but no direct connection between skull and hyoid bones. *Hapalops* lacks a stylohyoid process, instead showing a pit for articulation of the stylohyoid bone of the hyoid apparatus. This feature is located far posteriorly on the ventral cranial surface, also maximizing possible anteroposterior lingual excursion.

*Hapalops* possesses relatively large, round occipital condyles positioned farther under the occiput than in many larger sloths. This orientation provides great ability to flex the head on the neck, correlating with upright posture. The articulation is oriented so that the atlas and more posterior cervical vertebrae face posteroventrally, and may indicate that these sloths spent at least some of their time in a semivertical position; a necessity for arboreal or semiarboreal animals. This contrasts with the more dorsoventrally flattened posteriorly projecting occipital condyles in later megatheriids, such as *Eremotherium* and *Megatherium*; animals with more horizontal cervical vertebral articulations that were terrestrial. This capability would allow *Hapalops* to move its head from a position aligned anterior to the neck to nearly ninety degrees. Such head mobility would allow the sloth to feed while walking or standing quadrupedally, as well as while assuming a bipedal stance ( Scott, 1937: Figure 408).

**Muscular adaptations and correlations—**The main sloth masticatory muscles used in cutting vegetation are the superficial masseter and the medial pterygoid, and sloth ancestry explains their unique arrangements, bony attachments and lines of action. Mandibular movements are also effected by deep masseter and temporalis muscles in most herbivores, although generally of lesser importance ( Turnbull, 1970). In sloths, the attachments and lines of actions of the latter group have been reoriented to compliment superficial masseter and medial pterygoid movements. Although the superficial masseter is positioned more like that of *Bradypus* than *Choloepus*, because the ascending portion of the jugal is more posteriorly oriented, the line of action is more horizontal as is that of *Choloepus,* and the reconstruction of the same segment in *Paramylodon* (= *Glossotherium harlani* in Naples, 1989) ( Naples, 1982; Naples, 1985a; Naples, 1987). The line of action of the medial pterygoid in *Hapalops* is more horizontal than in the tree sloths, *Nothrotheriops*, and *Paramylodon* (= *Glossotherium*) ( Naples, 1982; Naples, 1985a; Naples, 1987; Naples, 1989). Specifically, the summed lines of action of superficial masseter and medial pterygoid are approximately 105 degrees to those of deep masseter, temporalis, and zygomaticomandibularis muscles ( Figure 8). In contrast, the average line of action in *Bradypus* is approximately 80 degrees, while that of *Choloepus* is approximately 100 degrees ( Naples, 1982).

Muscle reorientation turns the elongate cranium in *Hapalops* into an advantage by allowing a larger temporalis muscle with a greater arc of rotation, albeit with a more horizontal line of action. The significance of a more horizontal line of action for the temporalis, deep masseter, and zygomaticomandibularis muscles is an emphasis on the anteroposterior component of motion. The orientation for the line of action of temporalis is similar to that of *Choloepus* and *Paramylodon*, but less vertical than in *Bradypus* ( Naples, 1982; Naples, 1985a; Naples, 1989). The deep masseter orientation is approximately 45 degrees anterior to vertical, and reminiscent of the orientation in *Bradypus* and *Paramylodon*, but is relatively steeper than the more horizontal orientation in *Choloepus* and opposite to the posteriorly inclined muscle in *Nothrotheriops* ( Naples, 1982; Naples, 1985a; Naples, 1987; Naples, 1989). The zygomaticomandiularis line of action in *Hapalops* is more vertical than in any of the other sloths studied, but is not anteriorly oriented as in *Bradypus* ( Naples, 1982; Naples, 1985a; Naples, 1987; Naples, 1989). This arrangement sets these muscles in opposition to the increase of the vertical component of the line of action that would increase the mechanical advantage of the superficial masseter and medial pterygoid muscles. *Bradypus* has an increased the mechanical advantage for these muscles by elevation of the glenoid fossa and lowering of the insertion from the deepening of the mandibular ramus. Both *Bradypus* and *Hapalops* show posterior elongation of the mandibular angular process to a greater extent than in many other sloths. This feature and ventral pterygoid flange expansion allow an increased attachment area for the medial pterygoids, despite simultaneously increasing the anteroposterior component of mandibular motion. Such a mandibular motion in *Hapalops* would have become even greater if the craniomandibular joint remained at the level of the occlusal plane (approaching 120 degrees), but this angle has been reduced because this sloth has only slight craniomandibular elevation ( Figure 8A).

As active and balancing side masticatory muscles counteract lateral components of motion, superficial masseter and medial pterygoid muscle contraction with temporalis, deep masseter, and zygomaticomandibularis muscles cancel much of the anteroposterior component of motion. This results in mandibular elevation and allows a primarily mediolaterally oriented masticatory stroke. Mediolateral movements maximize the chewing stroke along the long axes of the teeth in *Hapalops*. Loss of a large anteroposterior component of motion, while decreasing masticatory efficiency, is less critical to *Hapalops* than other herbivores because of the large masticatory muscle mass and the small degree of gape. In *Hapalops* these are compensated during food ingestion by inheritance of a large, elongate tongue. Tongue usage was compensated for by the buccinator muscle, by allowing the mandible to be maintained in a slightly open position, such that the tongue could be rapidly and/or forcefully extended and retracted. The mylohyoid line is located in an unusually low position on the medial mandibular surface, confirming that there was a long and deep intraoral space. A long oral cavity is a liability offset by an elongated buccinator muscle. Large buccal cavities increase masticatory efficiency by accommodating and controlling the mediolateral movement of masses of vegetation during each masticatory cycle. Such an accommodation would also benefit *Hapalops*.

**Estimation of gape—**The degree to which an animal can gape correlates with the manner of feeding ( Herring & Herring, 1974; Herring, 1975). Sloths arose from insectivorous forms, and retain elongate crania and small buccal openings. Other factors that affect the masticatory pattern include the dental occlusal pattern and the shape, position and orientation of the mandibular condyle. Gape in *Hapalops* was estimated by comparison of the anterior and posterior maxima to which the condyles could rock in the glenoid fossa, and was found to be no more than 45 degrees. A wider opening would cause mandibular dislocation, and the angular processes to bump against neck tissues. Condylar convexity also permits some mediolateral rocking, providing the ability to swing the mandible labiolingually, as would be required to complete the lingual component of the anterolingual masticatory stroke indicated by the wear facet pattern in these sloths ( Greaves, 1973; Naples, 1990). The craniomandibular joint in *Hapalops* is only slightly elevated above the occlusal plane ( Figure 10A). This joint arrangement emphasizes movement in the mediolateral plane, as demonstrated by the shape and orientation of the dental occlusal facets ( Figure 4).

Given the cranial musculoskeletal arrangement of *Hapalops*, the initial masticatory cycle movement would have been mandibular elevation until dental occlusion occurred by combined masseter and temporalis muscle action. Because *Hapalops* only gapes to a small degree, mandibular elevation with a great force would have been possible. This degree of force is supported by an unpublished assessment of bite force ratios for a number of small-bodied sloths, including *Hapalops* (by RKM), which shows that the moment arm for M. masseter has the greatest effect upon mandibular elevation, and that the greatest bite forces occurred at the posterior dentition. Once the molariform teeth engaged, the mandible would complete the stroke cycle in a mediolateral direction before briefly depressing the jaw and repeating. The mediolaterally elongated glenoid fossa provided the space to allow the mandibular condyle to pivot, in the mediolateral swing provided by pterygoid muscles. Whereas the initial masticatory cycle movement could produce a suitable degree of crushing force, greater food processing would be achieved by grinding forces produced during the second half of the cycle. Because the greatest ratios for relative bite force were at the posterior dentition, as is common in herbivores, large initial forces would then be applied to the mediolaterally oriented grinding stroke.

## Conclusions

Adaptive shifts in the proto-sloth lineage from insectivory or myrmecophagy toward obligate herbivory resulted in a masticatory musculature rearrangement in *Hapalops* that reflects the typical herbivore pattern, although achieved by a unique combination of cranial musculoskeletal features. As the masticatory muscles and their cranial attachments were reduced in sloth ancestors that emphasized tongue-based food ingestion, sloths were free to develop these re-emphasized structures unconstrained by patterns inherited by other herbivores. Thus, in spite of the phylogenetic constraints of myrmecophagous ancestors, pilosans became successful, albeit atypical, herbivores. These features in *Hapalops* also occur in later megatheriids and other large-bodied sloths, and selective pressures resulting in the diversity of form in sloths are variations upon the theme of increased masticatory efficiency for herbivory. As such, the reconstruction of the cranial morphology and functions of *Hapalops* presented here can be used as a basis for understanding the selective pressures channeling the evolution of later megatheroids, and the relative contribution of phylogenetic history and functional constraints to the trend toward increased size and possible changes in locomotor habits and habitats.

**Figure 11.**
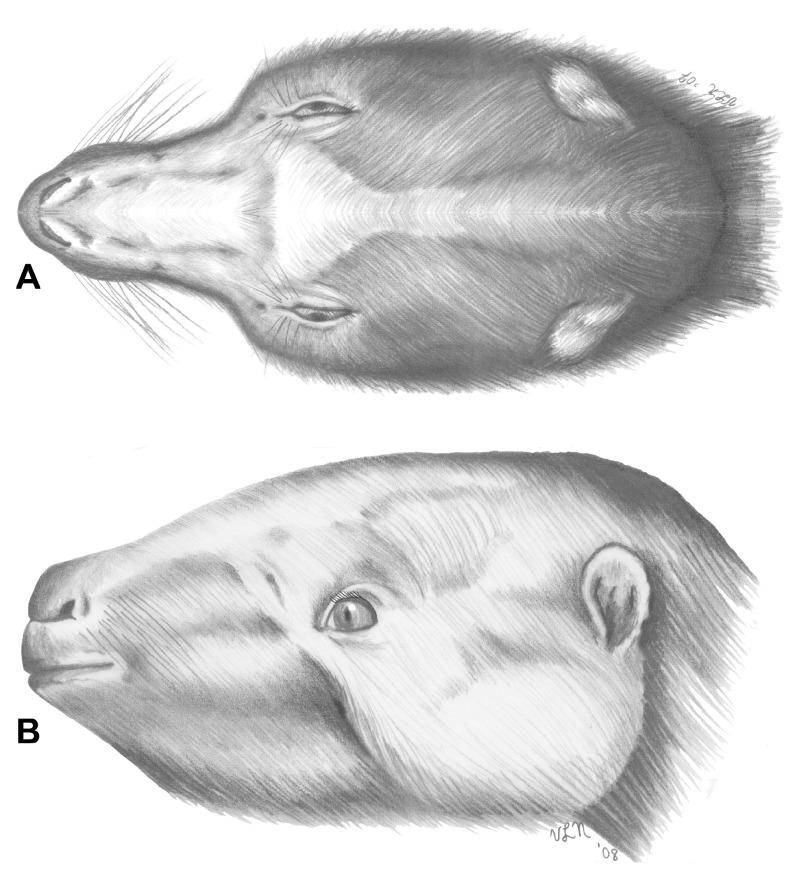
Reconstruction of the appearance of the head of *Hapalops* in dorsal view ( **A**), showing the narrowness of the muzzle, and the much greater posterior width of the head, and in lateral view ( **B**), showing the greatly elongated anterior portion of the cranium in this animal. This reconstruction results from new anatomical data generated in this study.

Reconstruction of cranial osteology and masticatory musculature demonstrates that the appearance of the head of *Hapalops* probably differed significantly from that of other small-bodied sloths. The zygomatic arches flare widely, making sloth heads wider at this location as well as longer than usually depicted ( Figure 1A, Figure 2, Figure 11). Likewise, the pointed anterior tips of the premaxilla and predental spout suggest an extreme narrowness of the anterior part of the muzzle, which was not appreciated in previous studies.
