# Supplementary material for: Chewing through the Miocene: an examination of the feeding musculature in the ground sloth Hapalops from South America (Mammalia: Pilosa)
Source: F1000Res. 2014 Apr 4;3:86. [Version 1] doi: 10.12688/f1000research.3282.1 (PMC4097365; doi:10.12688/f1000research.3282.1)
Supplement: Peer review history

# Peer review: Referee response for version 1

**DOI:** 10.5256/f1000research.3522.r4376

**Published:** 2014-06-11

**Authors:** David Burnham, Joshua Schmerge

**Affiliations:** 1 Biodiversity Institute, University of Kansas, Lawrence, KS, USA; 2 Department of Geology, University of Kansas, Lawrence, KS, USA

This is a very good paper and the science is sound. We suggest some minor technical writing issues that need to be addressed (see below). We think the figures could be much improved by making them less busy. Some suggestions were handled as comments, but overall many of the figures could be improved by shifting words out of the figure and into the captions or supporting text. The first two figures in particular have many words, and we think some of the labelled anatomical structures are either unnecessary to label or could be abbreviated to make the figure appear less busy. That being said, the figures are very good and help considerably in making the arguments outlined in the paper

Specifics:

- Page 2, First Paragraph – The second sentence reads “ *i.e., the extinct glyptodonts and giant armadillos, and the living armadillo*s”, instead it should read “i.e., the extinct glyptodonts, extinct giant armadillos, and the living armadillos”.
- Page 2, Fourth Paragraph – The seventh sentence (“ *Extinct sloths have been divided…*”) is redundant with the first paragraph of the introduction and should be rewritten.
- Page 2, Fourth Paragraph – In the eighth sentence, “ *small sized*” should be “small-sized”.
- Page 2, Seventh Paragraph – In the first sentence, it is not clear to what institution “ *Department of Geology”* refers. Furthermore, Table 1 contains a reference to YPM that is not explained in this paragraph.
- Page 2, Seventh Paragraph – In the second sentence, “adult” does not require quotation marks.
- Page 3, Table 1 – see previous comment about no explanation for the abbreviation YPM. It will also need to be added to the table caption.
- Page 4, Figure 1 – The label pointing out the zygomatic arch in Fig. 1A may be unnecessary for the intended audience, and removing the label will clean up the diagram. Furthermore, the label “Temporal” in the lower portion of Fig. 1A doesn’t seem to refer to anything since it lacks an arrow and it is not contained inside the line drawing.
- Page 7. Figure 4 – The sentence in the figure about the shaded surfaces indicating occlusion is also included in the figure caption and can be removed from the figure itself.
- Page 12, Figure 8 – I would prefer to read the explanations of the arrows in the figure caption rather than overlaying the figure.
- Page 13, Sixth Paragraph – The third sentence reads “ *The relative size, orientation and position of these *…” should read “The relative size, orientation, and position of these…”.
- Page 14, Seventh Paragraph – In the first sentence, “ *muscle*has” should be “muscle has”.
- Page 15, Twelfth Paragraph – The first sentence (“ *This muscle group controls the soft palate position and facilitates*.”) is an incomplete sentence.
- Page 16, Fifth Paragraph – The fourth sentence “ *The fossil record…*”  should be referenced.
- Page 17, First Paragraph – The first sentence ends with “ *denoting change in habitus*”. Is the word “habitus” really the word that was intended? I think the word habit would be preferable.

We have read this submission. We believe that we have an appropriate level of expertise to confirm that it is of an acceptable scientific standard.

# Peer review: Referee response for version 1

**DOI:** 10.5256/f1000research.3522.r4369

**Published:** 2014-05-06

**Authors:** Jennifer White

**Affiliations:** 1 Biology Department, East Stroudsberg University, East Stroudsburg, PA, USA

**Overall Impression** **:**

*Hapalops* is an important genus in sloth evolution: as a Miocene sloth it is old enough to have given rise to many later specialized genera of sloths, it exhibits cranial characters associated with both insectivory and herbivory, it is well represented in the fossil record, and there is substantial evidence that this genus was arboreal or semi-arboreal. Therefore, a detailed study of cranial morphology and inferred feeding habits is of great interest. This study parallels and expands upon previous studies by the first author on masticatory anatomy and function of other xenarthrans such as living tree sloths, the extinct sloths *Nothrotheriops* and *Glossotherium*, and living anteaters. The descriptive morphology is thorough, the illustrations are precise, the interpretations appear to be sound, and the conclusions are justified. Although the paper is well written, I have a number of comments and suggestions for minor revision outlined below.

**Abstract:**

- The phrasing in this sentence is awkward and should be revised: “ *An elevated temporomandibular joint occurs in Bradypus, a tree sloth with anterior chisel-shaped teeth instead of caniniforms, and the tree sloth Choloepus, which is aligned with the megalonychids, has anterior caniniforms*."

**Introduction** **:**

- The statement “ *Perhaps the young of even the largest species could climb*” is speculative; without evidence from fossil juvenile material why make a statement about it?
- White (1997) and White (2012) also presented evidence of arboreality in extinct sloths:

[White, J. L. 2012. Phylogenetic and functional diversity of West Indian sloths. In: R. Borroto-Paez, C. A. Woods, and F. E. Sergile (eds.), *Terrestrial* *Mammals of the West Indies; Contributions*. Gainesville, FL: Florida Museum of Natural History Press, pp. 317-336.]

[White, J. L. 1997. Locomotor adaptations in Miocene xenarthrans. In: R. F. Kay, R. H. Madden, R. L. Cifelli, J. J. Flynn (eds.), *Vertebrate Paleontology in the Neotropics: The Miocene Fauna of La Venta, Colombia*. Washington: Smithsonian Institution Press, pp. 246-264.]

- Muizon *et al.* 2004 (cited regarding *Thalassocnus*) does not appear in the reference list.
- The authors should reference Bargo *et al.* (2009), which provides an analysis of cranial morphology and feeding mechanics of *Eucholaeops*, another Miocene megatherioid sloth.

[Bargo *et al.*  2009. Predominance of orthal masticatory movements in the Early Miocene *Eucholaeops* (Mammalia, Xenarthra, Tardigrada, Megalonychidae) and other megatherioid sloths. *J. Vertebrate Paleontology* 29(3): 870-880.]

**Materials and Methods:**

- *Bradypus* and *Choloepus* were used to infer muscle position in *Hapalops*. These sloths have very different facial morphologies, so how was it determined which to use for comparison?
- The authors state “ *No Hapalops crania or mandibles were sufficiently complete or uncrushed to allow articulation*”. Table 1 lists four specimens with both skull and mandible; were these in such poor condition that they could not be articulated?
- An explanation of how the line of action of muscles was determined should be included in Methods.

**Results** **:**

- Figure 2: the caption has a spelling error in the word “osteological”.
- The statement “ *Animals with a farther lateral superior temporal line are probably younger, …*” is again speculative; is there other evidence to support a juvenile status, such as suture morphology?

Section on Mandibular Bony Features

- References should be provided for statements about sloth autapomorphies (under lingual and lateral view characters).
- Reiss (1997) discusses plesiomorphic states in the feeding apparatus for xenarthrans, along with derived conditions in myrmecophagids – perhaps this paper deserves to be cited.

[Reiss K.Z. 1997. Myology of the feeding apparatus of Myrmecophagid anteaters (Xenarthra: Myrmecophagidae). *J. Mammalian Evolution* 4(2): 87-117.]

- Regarding the section on mandibular lateral view characters: the mental nerve is a branch of the mandibular division of trigeminal (V _3_) which supplies motor innervation to muscles of mastication and carries sensory innervation from the lips; motor innervation to muscles of the lips (e.g. M. orbicularis oris, M. depressor labii inferioris) is supplied by the facial nerve.
- Regarding the section on mandibular dentition: a reference is needed for the statement that large interdental spaces are unusual in herbivores, and for the statement that unfused zygomatic arches distinguish pilosans from most other mammals.

Reconstruction of Cranial Musculature

- Figure 8: How is it determined exactly where lines of action of muscles are? Is this simply based on the apparent mid-point of each muscle scar?
- In the discussion of M. temporalis, the last sentence of the first paragraph should refer to Figure 10B (not 9A).
- A reference is needed for the statement about M. buccinatorius having many patterns among mammals.
- Figure 5: Where is the distinction between M. rectus capitis posterior major et minor? Is this muscle called M. rectus capitis *dorsalis* in the text, but *posterior* in the figure? 
- Figure 5: Does M. obliquus superioris (as labeled in the figure) represent M. obliquus capitis cranialis and caudalis (as referenced in text)?
- The first sentence under Palatine Musculature is incomplete.

**Discussion** **:**

- McAfee (2011) should be cited as the paper discusses characters associated with herbivorous feeding in sloths and derives bite force estimates.

[McAfee, R.K. 2011. Feeding mechanics and dietary implications in *Neocnus* (Mammalia) Xenarthra: Megalonychidae from Haiti. *J. Morphology* 272 (10): 1204-1216.]

- What is the functional significance of pterygoid flange extension?
- White (1997) [see citation above] also proposed arboreality in *Hapalops.*

Section on osteological adaptations and correlations:

- In the phrase “ *In many mammals, such as Choloepus and carnviorans,…*”, the word carnivorans is misspelled.
- The phrase *“…much weaker than that of the similarly sized buccinators, Nematherium” – *is unclear. What is meant by “buccinators”?

Section on osteological adaptations and correlations:

- I question how accurate the estimate of 105 degrees (summed lines of action of superficial masseter and medial pterygoid) is when it is unclear how the lines of actions of muscles were derived. Also, what is the functional significance of this value for these muscles in *Hapalops* in comparison to those of the living tree sloths?
- It is difficult to evaluate the validity of the bite force estimates in *Hapalops* that are derived from unpublished analyses by McAfee. 

I have read this submission. I believe that I have an appropriate level of expertise to confirm that it is of an acceptable scientific standard.
